# Combined Omics Approaches Reveal Distinct Mechanisms of Resistance and/or Susceptibility in Sugar Beet Double Haploid Genotypes at Early Stages of Beet Curly Top Virus Infection

**DOI:** 10.3390/ijms241915013

**Published:** 2023-10-09

**Authors:** Paul J. Galewski, Rajtilak Majumdar, Matthew D. Lebar, Carl A. Strausbaugh, Imad A. Eujayl

**Affiliations:** 1 Northwest Irrigation and Soils Research Laboratory, United States Department of Agriculture—Agricultural Research Service, Kimberly, ID 83341, USA; paul.galewski@usda.gov (P.J.G.); carl.strausbaugh@usda.gov (C.A.S.); imad.eujayl@usda.gov (I.A.E.); 2 Plant Germplasm Introduction and Testing Research Unit, United States Department of Agriculture—Agricultural Research Service, Pullman, WA 99164, USA; 3 Food and Feed Safety Research Unit, Southern Regional Research Center, United States Department of Agriculture—Agricultural Research Service, New Orleans, LA 70179, USA; matthew.lebar@usda.gov

**Keywords:** BCTV (beet curly top virus), sugar beet, KDH13, KDH4-9, KDH19-17, virus, host resistance, RNAi, non-coding RNAs, small ORFs, small peptides, flavonoids, isoflavonoids, triterpenoids, RNAseq, degradome, peptidome, untargeted metabolome

## Abstract

Sugar beet is susceptible to Beet curly top virus (BCTV), which significantly reduces yield and sugar production in the semi-arid growing regions worldwide. Sources of genetic resistance to BCTV is limited and control depends upon insecticide seed treatments with neonicotinoids. Through double haploid production and genetic selection, BCTV resistant breeding lines have been developed. Using BCTV resistant (R) [KDH13; Line 13 and KDH4-9; Line 4] and susceptible (S) [KDH19-17; Line 19] lines, beet leafhopper mediated natural infection, mRNA/sRNA sequencing, and metabolite analyses, potential mechanisms of resistance against the virus and vector were identified. At early infection stages (2- and 6-days post inoculation), examples of differentially expressed genes highly up-regulated in the ‘R’ lines (vs. ‘S’) included *EL10Ac5g10437* (inhibitor of trypsin and hageman factor), *EL10Ac6g14635* (jasmonate-induced protein), *EL10Ac3g06016* (ribosome related), *EL10Ac2g02812* (probable prolyl 4-hydroxylase 10), etc. Pathway enrichment analysis showed differentially expressed genes were predominantly involved with peroxisome, amino acids metabolism, fatty acid degradation, amino/nucleotide sugar metabolism, etc. Metabolite analysis revealed significantly higher amounts of specific isoflavonoid O-glycosides, flavonoid 8-C glycosides, triterpenoid, and iridoid-O-glycosides in the leaves of the ‘R’ lines (vs. ‘S’). These data suggest that a combination of transcriptional regulation and production of putative antiviral metabolites might contribute to BCTV resistance. In addition, genome divergence among BCTV strains differentially affects the production of small non-coding RNAs (sncRNAs) and small peptides which may potentially affect pathogenicity and disease symptom development.

## 1. Introduction

Beet curly top virus (BCTV) is an important member belonging to the family *Geminiviridae* and genus *Curtovirus*, which infects a wide variety of plant species including commercially important crops, such as common beans, cucurbits, hemp, pepper, spinach, sugar beet, and tomatoes [1,2,3,4]. The virus poses a significant threat to sugar beet (*Beta vulgaris* L.) production in semi-arid regions across the globe and thus negatively affects sugar production. In the United States in 2021, 54% of the sugar produced came from sugar beet (United States Dept. Agric.-Nat. Agric. Stat. Service). Mitigating BCTV mediated crop losses in sugar beet primarily depends upon controlling the beet leafhopper (BLH; *Circulifer tenellus* Baker) through the use of neonicotinoid insecticide seed treatments but the potential for resistance to buildup and non-target control issues are both concerning [5,6,7]. Host resistance offers the most economical means to alleviate BCTV problems, but currently commercial sugar beet cultivars only contain low to intermediate levels of resistance [4,6,8]. Therefore, new sources of genetic resistance to BCTV and a higher level of resistance is greatly desired. Advances in genomics and functional genomics offer cutting-edge tools to investigate the landscape of host–virus interactions, which may lead to additional management options in sugar beet.

Plant defense strategies against pathogens including viruses and pests are multilayered and occur at the level of transcription, translation, and metabolites [9,10,11]. Host plant responses to both DNA and RNA viruses and changes in global gene expression and/or protein levels have been studied in multiple crop species including potato (*Solanum tuberosum* L.), Chinese cabbage (*Brassica rapa* ssp. *pekinensis*), sugarcane (*Saccharum* spp.), melon (*Cucumis melo* L.), pecan (*Carya illinoinensis*), etc. [12,13,14,15,16]. Besides common virus targets in host plants such as genes associated with virus replication/transcription/translation, phytohormone pathways, and RNA silencing related to antiviral pathways, the specific key pathways in defense against a specific viral pathogen may vary depending upon crop species and whether the virus tested was insect transmitted. In potatoes defending against the potato virus Y (PVY; RNA virus), photosynthesis-related genes were most impacted at both protein and transcript levels [12]. In melons, differentially expressed genes (DEGs) in response to the tomato leaf curl New Delhi virus (ToLCNDV) were associated with the jasmonic acid (JA) signaling pathway, photosynthesis, RNA silencing, the transmembrane, and sugar transporters [15]. In pecans (*Carya illinoinensis*), there is up-regulation of *pathogenesis*-*related protein* 1 (*PR*1) and down-regulation of *phenylalanine ammonia*-*lyase* (*PAL*) and *isochorismate synthase* (*ICS*) during pecan mosaic virus (PMV; DNA virus) infection, indicating a putative role of salicylic acid (SA) biosynthetic pathway in host plant defense against the virus [16]. Putative roles of plant derived secondary metabolites (SMs) in resistance against viruses have been reported under diverse plant–virus interactions, though the exact function of specific SMs remains to be elucidated [9,10,17]. The wide range of SMs produced by plants in response to biotic stresses, including virus infections, primarily belong to three major groups including terpenes/terpenoids, phenolic compounds (such as flavonoids and phenolic/polyphenolic compounds), and nitrogen/sulfur-containing compounds (such as alkaloids and glucosinolates). Many of these SMs have been implicated in host plant resistance against viruses.

Small RNAs have been shown to play critical roles in host plant resistance against pathogens and insect feeding [18,19]. The role of small non-coding RNAs (sncRNAs) and micro RNAs (miRNAs) in BCTV resistance using resistant/susceptible sugar beet genotypes have been reported earlier [20]. Yet major questions regarding the complexities of these systems remain in understanding how different strains of the virus affect virulence in different sugar beet genotypes. Besides sncRNAs, small peptides originating from putative small open reading frames (sORFs) in Geminiviruses have also been implicated in diverse functions such as nuclear localization and their putative roles in virulence [21,22]. The availability of genome sequences of BCTV strains relevant to sugar beet production in the western U.S. have added to our understanding on prevalent strains [6]. Our ability to investigate the divergence among virus strains with respect to function will be critical in identifying potential viral pathogenicity factors, their host target genes, and testing their putative roles in virulence.

Pathogen strain specific responses of host plants are complex [23], which is especially true in viruses where some virus strains produce strong disease symptoms while others do not. While genetic composition of host plants plays a key role, viral pathogenicity factors such as sncRNAs can act as modulators of host gene expression through cross-kingdom RNA interference (RNAi) and affect host resistance [24]. Different BCTV strains in sugar beet and their relative abilities to produce disease symptoms in diverse genotypes have been reported in earlier studies [6,25,26]. The predominant BCTV strains found in sugar beets grown in the western U.S. include California/Logan (CA/Logan) strain CTS06-104 (GenBank accession KX867032.1), Colorado (CO) strain CTS15-113 (KX867056.1), Severe (Svr) strain CTS06-021 (KX867019.1), and Worland (Wor) strain CTS15-095 (KX867055.1). Based upon available data, Svr and CA/Logan strains have been the most virulent in sugar beet, but our knowledge of how these strains produce more severe symptoms is limited [6]. We do not fully understand how genetic divergence among BCTV strains might lay the precedence for future mutations in the genome thereby affecting pathogenicity. How sugar beet plants respond to BCTV infection at the level of global gene expression and produce metabolites that could potentially contribute to host plant resistance is unknown. In addition, the role of viral pathogenicity factors, namely sncRNAs originating from different BCTV strains, and the putative role of virus-derived small peptides in the development of disease symptoms are not fully understood. Previously, the sncRNAs originating from the BCTV severe (Svr) strain and their putative roles were investigated [20]. In this study, sncRNAs from different BCTV strains and their putative interactions with sugar beet target genes in a strain specific manner using degradome analysis have been investigated. Better understanding of early events in host plants in response to viruses and their contribution towards resistance and/or susceptibility will help in designing future mitigation strategies. A system biology approach using multi-omics tools was taken to dissect early events during sugar beet–BCTV interactions. Using BCTV resistant/susceptible sugar beet double-haploid genotypes developed by our research group and a combination of tools including global RNA sequencing, metabolite, degradome, and viral peptidome analyses, the potential mechanisms of BCTV resistance/susceptibility were demonstrated. The sugar beet double haploid and homozygous ‘R’ lines (Line 13 and Line 4) and the ‘S’ line (Line 19) used in this study originated from independent events. The ‘R’ lines were created from the sugar beet population C762-17 (PI560130) and the ‘S’ line originated from the C944 population (PI663873). Both ‘R’ and ‘S’ lines used in this study have been tested for BCTV resistance/susceptibility under field conditions across multiple growing seasons [27,28,29]. The results presented here will have future implications towards the development of virus resistant sugar beet cultivars.

## 2. Results

### 2.1. Differentially Expressed Sugar Beet Genes at Early Stages of BCTV Infection

**Figure 1:**
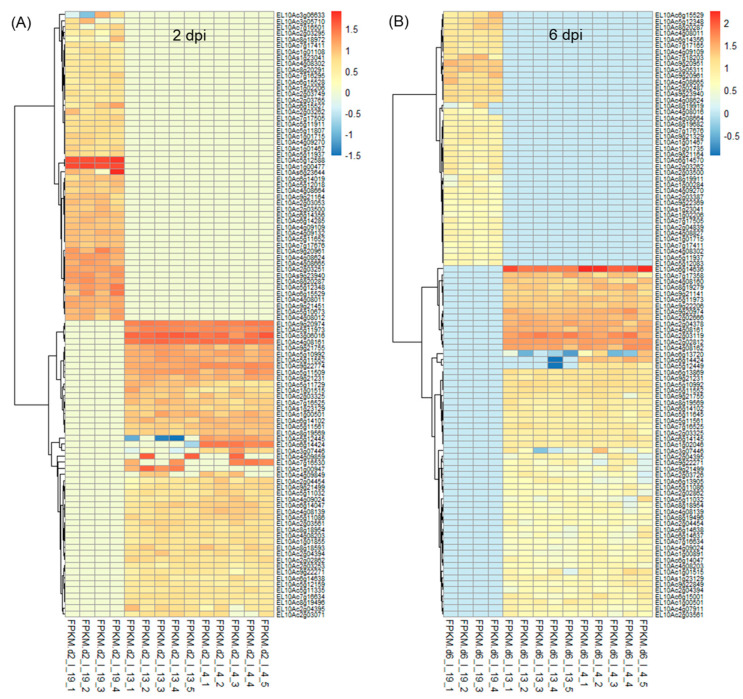
Heatmaps showing a subset of differentially expressed (DE) transcripts (*p* < 0.01) at (**A**) 2 days post inoculation (dpi), and (**B**) 6 dpi in the leaves of Beet curly top virus susceptible (Line 19; S) and resistant (Line 13 and Line 4; R) sugar beet lines. Data are Mean ± SE of 4–5 biological replicates and *p* < 0.01 between ‘S’ and ‘R’ lines; I = infected].

On average, ~43 million raw reads/sample and ~42 million valid reads/sample were obtained (Table S1). The total number of differentially expressed (DE) genes at 2 dpi and 6 dpi along with uninfected control samples at those time points are shown in Figure S1. At 2 dpi, a total number of 8459–8464 differentially expressed transcripts were found when compared between ‘R’ and ‘S’ lines (Figure S1A). Amongst which, 163 transcripts were unique to Line 13, and 158 transcripts were unique to Line 4. The total number of differentially expressed transcripts was lower (5460–5462) in the 2 d control plants between ‘R’ and ‘S’ (Figure S1B) where 152 transcripts were unique to Line 13, and 154 transcripts were unique to Line 4. The total number of differentially expressed transcripts (5275–5276) was lower at 6 dpi (vs. 2 dpi) between ‘R’ and ‘S’, where 182 transcripts were unique to Line 13, and 181 transcripts were unique to Line 4 (Figure S1C). The 6 d control plants showed 197 unique differentially expressed transcripts in the ‘R’ lines and a total of 4447 differentially expressed transcripts, when compared between ‘R’ and ‘S’ lines (Figure S1D). Heatmaps of DE genes (*p* < 0.01) at 2 and 6 dpi are shown in Figure 1 and the control samples in Figure S2. All DE genes (*p* < 0.05) in the infected (BCTV/BLH fed) and uninfected plants from the ‘S’ and ‘R’ lines at 2 d and 6 d are presented in Tables S2–S5. At 2 days post inoculation, (dpi) genes that were highly up-regulated (Figure 1A; Table S2) only in the ‘R’ lines (Line 13, Line 4) with very little to no expression (mean FPKM < 1) in the ‘S’ line in most cases included *EL10Ac3g06016* (ribosome related; mean FPKM: 31–37), *EL10Ac4g08161* (late embryogenesis abundant protein; mean FPKM: 31), *EL10Ac9g20974* (unknown; mean FPKM: ~26), *EL10Ac5g11973* (protein FIZZY-RELATED 3-like isoform X5; mean FPKM: 21), *EL10Ac9g21231* (SPX domain-containing membrane protein; mean FPKM: 11–12), etc. Examples of genes that were moderately expressed in the ‘S’ line but were highly up-regulated (>65 to 217-fold) in the ‘R’ lines included *EL10Ac5g10437* (inhibitor of trypsin and hageman factor; mean FPKM: 1427–2198 vs. mean FPKM: 22 in ‘S’), *EL10Ac6g14635* (jasmonate-induced protein; mean FPKM: 416–433 vs. mean FPKM: 2 in ‘S’), etc. At 2 dpi, genes that were highly up-regulated only in the Line 4 with no expression in the ‘S’ line and with very little expression (mean FPKM < 1) in the Line 13 in most cases included *EL10Ac5g12445* (F-box protein; mean FPKM: 14), *EL10Ac6g14424* (MAPK signaling pathway related; mean FPKM: 26), *EL10Ac7g16530* (polyubiquitin; mean FPKM: 15), etc. Whereas *EL10Ac1g00947* (unknown; mean FPKM: 18) was only expressed in Line 13.

At 2 dpi, examples of genes highly expressed only in the ‘S’ line and with no expression in either of the two ‘R’ lines included *EL10Ac5g12588* (unknown protein; mean FPKM: 54), *EL10Ac9g20961* (phospholipase A1-IIdelta; mean FPKM: 16), etc. Whereas expression of *EL10Ac6g15504* (mannose/glucose-specific lectin isoform) was >18 to 22-fold in the ‘S’ line (mean FPKM: 5912) vs. ‘R’ lines (mean FPKM: 273–337).

At 6 dpi highly up-regulated genes in both ‘R’ lines with no expression in the ‘S’ lines (Figure 1B; Table S3) included *EL10Ac6g14636* (jasmonate-induced protein; mean FPKM: 74–135), *EL10Ac2g03119* (unknown; mean FPKM: 42–49), *EL10Ac2g02812* (probable prolyl 4-hydroxylase 10; mean FPKM: 37–39), *EL10Ac4g08162* (putative 1-phosphatidylinositol-3-phosphate 5-kinase FAB1D; mean FPKM: 32–36), etc. At 6 dpi an example of a gene highly up-regulated only in Line 4 with no expression in the ‘S’ line and very little expression (mean FPKM < 0) in Line 13 included *EL10Ac6g14424* (MAPK signaling pathway related; mean FPKM:24).

Some of the highly up-regulated genes only in the ‘S’ line with no expression in the ‘R’ lines at 6 dpi included *EL10Ac9g20951* (unknown; mean FPKM: 21), *EL10Ac9g20961* (phospholipase A1-IIdelta; mean FPKM: 17), *EL10Ac7g18203* (disease resistance protein; mean FPKM: 16), etc.

Differentially expressed genes in the uninfected control (C) treatments at 2 d and 6 d are presented in Figure S2. Examples of genes highly up-regulated only in the ‘R’ lines with little or no expression (mean FPKM < 1) in the ‘S’ line in the 2 d (Figure S2A; Table S4) samples included *EL10As2g23270* (alanine—glyoxylate aminotransferase 2 homolog 3, mitochondrial; mean FPKM: 64–75), *EL10As5g23570* (alanine--glyoxylate aminotransferase 2 homolog 3, mitochondrial; mean FPKM: 57–64), *EL10Ac6g13092* (ras-related protein RAB1BV-like; mean FPKM: 38–60), etc. Whereas genes highly up-regulated only in the ‘S’ line (vs. ‘R’ lines) included *EL10As5g23536* (photosystem I reaction center subunit V, chloroplastic; mean FPKM: 619 and up-regulated by 101 to 114-fold), *EL10Ac2g04748* (unknown; mean FPKM: 404 and up-regulated by 14 to 15-fold), etc.

At 6 d (Figure S2B; Table S5) genes that were highly up-regulated only in the ‘R’ lines (vs. ‘S’) in control samples included *EL10Ac6g14641* (jasmonate-induced; mean FPKM: 159–175 and up-regulated by to 834 to 919-fold), *EL10Ac3g06086* (70 kDa peptidyl-prolyl isomerase; mean FPKM: 178–206 and up-regulated by to 414 to 480-fold), *EL10Ac6g14635* (jasmonate-induced; mean FPKM: 409–521 and up-regulated by to 309 to 393-fold), etc.

Candidate genes highly up-regulated only in the ‘S’ line (vs. ‘R’ lines) at 6 d control samples included *EL10As5g23536* (photosystem I reaction center subunit V, chloroplastic, chloroplastic; mean FPKM: 581 and up-regulated by 105 to 154-fold), *EL10Ac3g05482* (jasmonate-induced; mean FPKM: 1390 and up-regulated by 46 to 75-fold), *EL10Ac3g05481* (jasmonate-induced; mean FPKM: 856 and up-regulated by 45 to 67-fold), etc.

### 2.2. GO and KEGG Analyses of Differentially Expressed Genes

**Figure 2:**
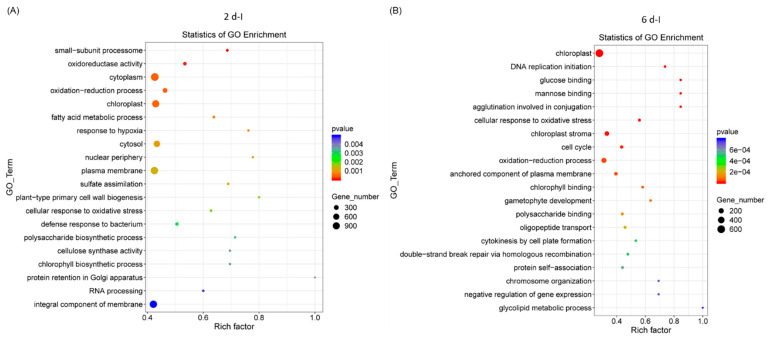
Gene ontology (GO) of differentially expressed sugar beet genes in the leaves of Beet curly top virus infected sugar beet plants. (**A**) 2 dpi and (**B**) 6 dpi. Data are mean of 3 biological replicates.

Gene ontology (GO) analysis of sugar beet genes that were differentially expressed, highly significant, and represented in higher numbers in the 2 dpi samples (Figure 2A) were primarily associated with cytoplasm, chloroplast, oxidation reduction, etc. At 6 dpi, DE genes were primarily related to chloroplast, oxidation-reduction, anchored component of plasma membrane, and cell cycle (Figure 2B). Examples of genes in the 2 d control (C) samples were related to the cytosolic large ribosomal subunit, the structural constituent of ribosome, cell cycle, etc., (Figure S3A) and in 6 d ‘C’ plants genes were primarily associated with plasma membrane, protein serine/threonine kinase activity, and defense response (Figure S3B).

**Figure 3:**
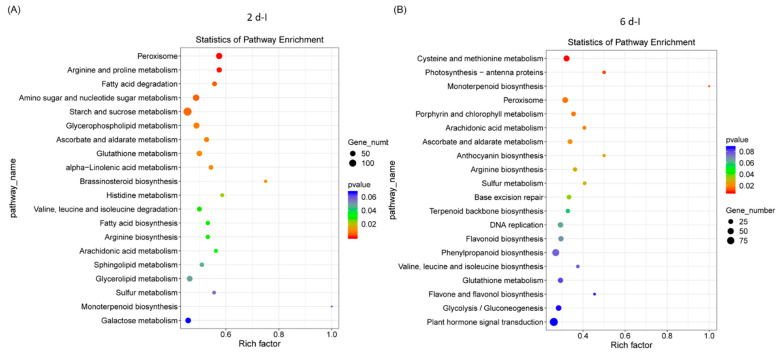
Pathway enrichment of differentially expressed sugar beet genes in the leaves of Beet curly top virus infected plants. Kyoto Encyclopedia of Genes and Genomes (KEGG) enrichment of sugar beet genes in (**A**) 2 dpi and (**B**) 6 dpi. Data are mean of 4–5 biological replicates.

Pathway enrichment analysis of differentially expressed sugar beet genes that were highly significant and represented in higher numbers in the 2 dpi plants were primarily related to peroxisome, arginine and proline metabolism, fatty acid degradation, amino sugar and nucleotide sugar metabolism, and starch and sucrose metabolism (Figure 3A). At 6 dpi, genes associated with pathways pertaining to cysteine and methionine metabolism, peroxisome, porphyrin and chlorophyll metabolism, ascorbate and aldarate metabolism, etc., were predominant (Figure 3B). In the control plants at 2 d, pathways were predominantly associated with ribosome, amino sugar and nucleotide sugar metabolism, arachidonic acid metabolism, arginine, and monoterpenoid biosynthesis (Figure S4A) and at 6 d, pathways associated with flavonoid biosynthesis, glutathione metabolism, arginine biosynthesis, and starch and sucrose metabolism were prevalent (Figure S4B).

### 2.3. WGCNA Analyses of Differentially Expressed Genes

**Figure 4:**
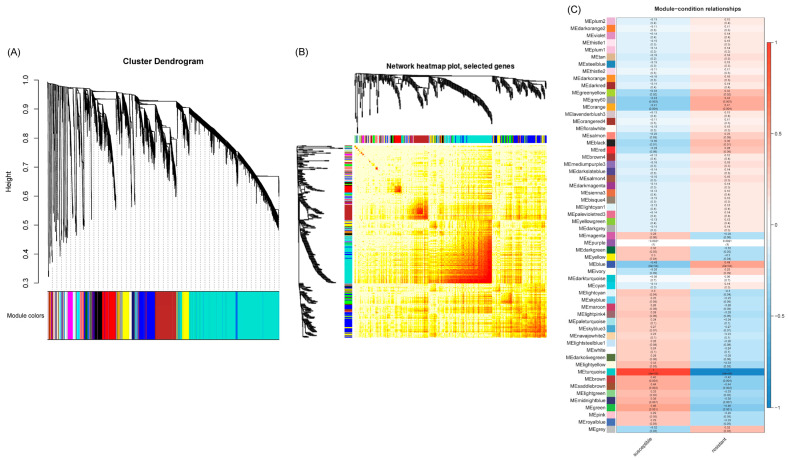
Weighted gene co-expression network analysis (WGCNA) of sugar beet genes in the leaves infected with or without Beet curly top virus (BCTV) show distinct clustering pattern in the BCTV susceptible and resistant sugar beet lines. (**A**) Gene cluster dendrogram, (**B**) Heatmap showing network of differentially expressed genes, and (**C**) module-sample relationship. Data are Mean of 4–5 biological replicates.

A weighted gene co-expression network analysis (WGCNA) was conducted using the count data from RNAseq reads that mapped to the sugar beet genome to establish a pairwise correlation between genes across samples. Correlation coefficients for all genes were determined along with the construction of a hierarchical clustering using the correlation matrices. Different colors represent different gene modules, and each module consists of multiple genes. The gene cluster dendrogram and the heatmap of sugar beet genes are shown in Figure 4A and Figure 4B, respectively. A highly co-expressed (positive correlation: 1; significance of *p* < 0.05) gene module that was evident only in the BCTV susceptible Line 19, both in the absence of the virus and with BCTV infection, is represented by MEturquoise (Figure 4C and Figure S5). Examples of candidate genes belonging to this module that showed high connectivity (>1000) are *EL10Ac8g20340* (hypothetical protein), *EL10Ac8g20339* (hypothetical protein), *EL10Ac2g03213* (protoporphyrinogen oxidase, chloroplastic/mitochondrial), *EL10Ac5g11959* (AP-4 complex subunit mu), *EL10Ac6g13933* (splicing factor U2af large subunit B), *EL10Ac2g04578* (oxidation resistance protein 1), *EL10Ac6g14270* (MATE efflux family protein 8), etc. The gene module representing genes that were moderately co-expressed (positive correlation: 0.41–0.48; significance of *p* < 0.05) in the resistant lines following infection included MEblue and MEgrey60. The module MEblue was consistent in the resistant Line 4 representing several candidate genes that showed moderate connectivity (>323–350) included *EL10Ac3g07378* (brassinosteroid insensitive 1-associated receptor kinase 1), *EL10Ac8g19281* (eukaryotic membrane protein of unknown function (DUF872), *EL10Ac3g05673* (membrane steroid-binding protein 2), *EL10Ac2g02491* (probable protein phosphatase 2C 60), *EL10Ac2g02436* (probable protein phosphatase 2C 59), *EL10Ac6g13903* (probable magnesium transporter NIPA8), etc. The modules MEorange and MEgrey60 were detected only in the resistant Line 13 following infection. Examples of a few of the top candidates belonging to the MEorange module are *EL10Ac9g22911* (organic cation/carnitine transporter 2), *EL10Ac9g22672* (NAD-dependent protein deacetylase SRT1), *EL10Ac9g22628* (zinc finger CCCH domain-containing protein 20), etc. Some of the top candidate genes in MEgrey60 module included *EL10Ac6g14628* (protein PHYLLO, chloroplastic), *EL10Ac6g13095* (DEAD-box ATP-dependent RNA helicase ISE2, chloroplastic), *EL10Ac8g20415* (peroxisome biogenesis protein 6), *EL10Ac2g04850* (anaphase-promoting complex subunit 1), *EL10Ac7g18133* (helicase SEN1), *EL10Ac6g14174* (probable ion channel SYM8), *EL10Ac8g19444* (nuclear pore complex protein NUP1), etc.

### 2.4. Metabolome Analysis Reveal Disctinct Differences between Resistant and Susceptible Lines at Early Infection Stages 

**Figure 5:**
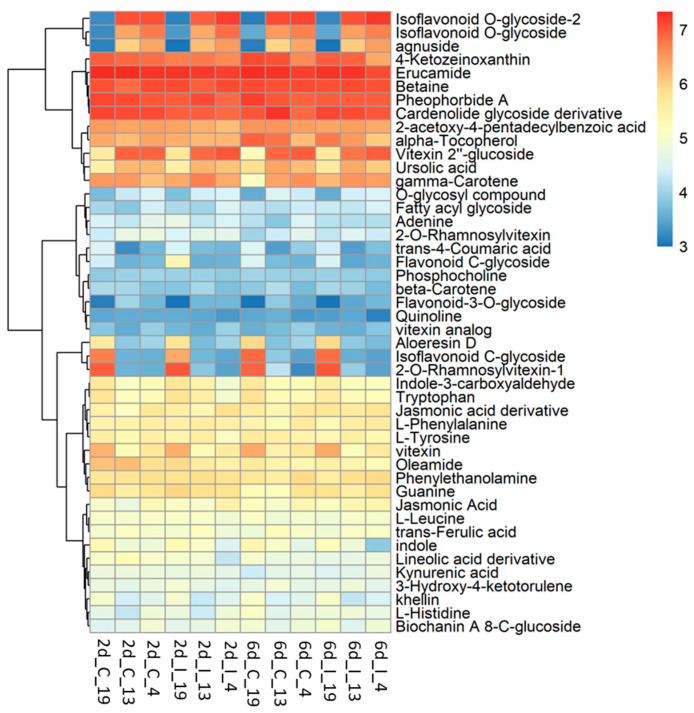
Heatmap of metabolites in the leaves of BCTV susceptible (Line 19; S) and resistant (Line 13 and Line 4; R) sugar beet lines at 2 d and 6 d in control (C; uninfected) and infected (I) samples. Data are Mean ± SE of 4–6 biological replicates.

Untargeted metabolomic analysis was conducted on the methanolic extracts of infected sugar beet leaf samples. Unlike metabolomic techniques utilizing data-dependent acquisition (DDA), untargeted metabolomics from data-independent acquisition (DIA) LC-MS/MS experiments require deconvolution of high and low energy mass spectra. DIA has the advantage of collecting high and low energy mass data for all precursor ions but comes with the disadvantage of producing very large and complex data sets. MS-DIAL [30] was used for deconvolution, peak picking, and alignment of DIA data sets from three sugar beet lines (‘S’: 19; ‘R’: 13, 4) under two treatments (C, I) at two time points (2d, 6d). Analysis of four to six replicates of each condition resulted in detection of more than 60,000 unknown peaks. About 35,000 peaks were correlated to metabolites using exact mass, however less than 100 of these could be verified with reference matched MS/MS spectra. Our analysis focused on 46 of these referenced match metabolites that had a high match score (>0.70) and an S/N average >5 (Figure 5; Table S6). 

**Figure 6:**
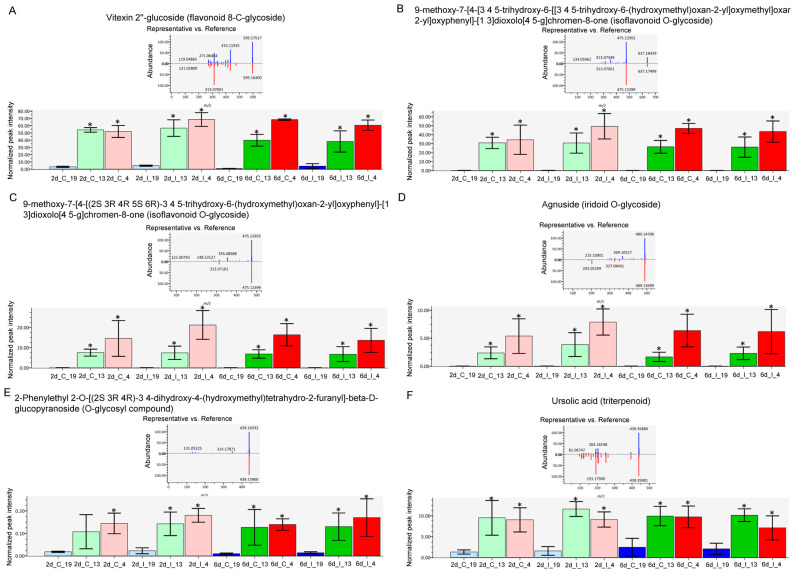
Specific metabolites highly abundant in the leaves of Beet curly top virus (BCTV) susceptible [Line 19; S (represented in blue color)] and resistant [Line 13 (represented in green color) and Line 4 (represented in pink color); R] sugar beet lines at 2 d and 6 d in control (C; uninfected) and infected (I) samples. (**A**) vitexin 2″-glucoside, (**B**) 9-methoxy-7-[4-[3 4 5-trihydroxy-6-[[3 4 5-trihydroxy-6-(hydroxymethyl)oxan-2-yl]oxymethyl]oxan-2-yl]oxyphenyl]-[1 3]dioxolo [4 5-g]chromen-8-one, (**C**) 9-methoxy-7-[4-[(2S 3R 4R 5S 6R)-3 4 5-trihydroxy-6-(hydroxymethyl)oxan-2-yl]oxyphenyl]-[1 3]dioxolo [4 5-g]chromen-8-one, (**D**) Agnuside, (**E**) 2-Phenylethyl 2-O-[(2S 3R 4R)-3 4-dihydroxy-4-(hydroxymethyl)tetrahydro-2-furanyl]-beta-D-glucopyranoside, and (**F**) ursolic acid. Data are Mean ± SE of 4–6 biological replicates; * *p* < 0.05 between ‘S’ and ‘R’ lines at a specific time point within uninfected control (C) or infected (I) samples.

The metabolites which showed the most difference between the ‘R’ lines and ‘S’ line in the leaves, and were much higher in the R lines, were primarily flavonoid and isoflavonoid derivatives, O-glycosides, and triterpenoid (Figure 6). These included vitexin 2″-glucoside (Flavonoid 8-C-glycoside), 9-methoxy-7-[4-[3 4 5-trihydroxy-6-[[3 4 5-trihydroxy-6-(hydroxymethyl)oxan-2-yl]oxymethyl]oxan-2-yl]oxyphenyl]-[1 3]dioxolo[4 5-g]chromen-8-one (Isoflavonoid O-glycoside), 9-methoxy-7-[4-[(2S 3R 4R 5S 6R)-3 4 5-trihydroxy-6-(hydroxymethyl)oxan-2-yl]oxyphenyl]-[1 3]dioxolo[4 5-g]chromen-8-one (Isoflavonoid O-glycoside), Agnuside (Iridoid O-glycoside), 2-Phenylethyl 2-O-[(2S 3R 4R)-3 4-dihydroxy-4-(hydroxymethyl)tetrahydro-2-furanyl]-beta-D-glucopyranoside (O-glycosyl compound), and ursolic acid (Triterpenoid). The majority of these compounds were higher in the ‘R’ lines at the basal level (in absence of BCTV) and virus infection did not significantly alter their cellular contents. A complete list of compounds detected in our study, that were differentially regulated in the ‘R’ vs. ‘S’ lines, are presented in Table S6. Some examples of metabolites that were much higher only in the ‘S’ line (vs. ‘R’ lines; Figure 5; Table S6) included 2-O-Rhamnosylvitexin (flavonoid 8-C-glycoside), 8-[3 5-dihydroxy-6-(hydroxymethyl)-4-(3 4 5-trihydroxyoxan-2-yl)oxyoxan-2-yl]-5 7-dihydroxy-3-(4-hydroxyphenyl)chromen-4-one (isoflavonoid C-glycoside), aloeresin D (phenolic glycoside), and vitexin (flavonoid 8-C-glycoside). BCTV infection did not significantly alter these metabolites in the ‘S’ line in comparison to the uninfected control plants.

### 2.5. BCTV Strain Specific sncRNAs and Their Interaction with Putative Sugar Beet Target Genes

**Figure 7:**
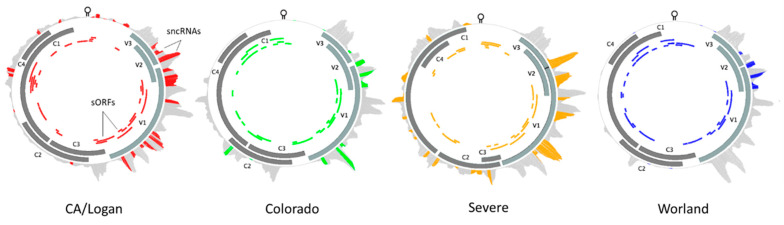
Beet curly top virus (BCTV) strain specific distribution and relative abundance of virus-derived small non-coding RNAs during sugar beet infection. The solid gray blocks and colored blocks denote main open reading frames (ORFs) and predicted small ORFs, respectively. The outer track, which is gray, denotes viral derived small non-coding RNAs, while the colored portion represents small non-coding RNA core sequences showing sequence complementarity to putative sugar beet target genes [V3: movement protein; V2: SS-DS DNA regulator; V1: capsid protein; C3: replication enhancer; C2: pathogenesis enhancement protein; C1: rolling circle replication initiator protein; C4: cell cycle regulator].

A single genome was chosen to represent each of the four BCTV strains of interest: CA/Logan (GenBank accession: KX867032.1), CO (KX867056.1), Svr (KX867019.1), and Wor (KX867055.1). Reads representing BCTV sncRNA extracted from the resistant and susceptible sugar beet genotypes showed preferential alignment to the four virus strain reference genomes. Strain specific differences in sncRNAs and putative sORFs were detected based on sequence alignment and identity (Figure 7, Table 1). Known ORFs which included all seven primary BCTV genes were filtered out from these sORFs and treated separately. The length and the number of putative sORFs among different BCTV strains varied between 17–212 bp and 25–31 ORFs, respectively (Figure 7, Table S10). The Wor strain contains the highest (38) and the Svr strain contains the lowest (32) number of sORFs. The distribution of the sORFs across BCTV strains showed both conserved (between CO and Wor) and distinct (in CA/Logan) patterns among strains. In total, 37 putative sncRNAs were detected across the genomes of four BCTV strains. Among detected sncRNAs, some were common to all four BCTV strains such as sncRNA_2, sncRNA_26, sncRNA_36, and sncRNA_37. Examples of strain specific sncRNA included sncRNA_16, sncRNA_20, and sncRNA_3 that correspond to Svr, CA/Logan, and CO strains, respectively. In some cases, sncRNAs were shared by two strains, for example sncRNA_19 was shared by CA/Logan and Svr strains.

The sncRNA sequences were used to identify putative sugar beet target genes using the EL10.1 reference genome. The mRNA expression levels of specific host target genes of BCTV sncRNAs were compared between the resistant (Line 13, Line 4) and susceptible (Line 19) genotypes to test genotype specific responses against BCTV strains. The correlation between the relative abundance of sncRNAs among host genotypes and putative sugar beet target gene expression allowed us to prioritize sncRNAs that had the most effect on sugar beet target genes (Table 2). Among the virus strains, CA/Logan, CO, and Svr contained three sncRNAs and Wor contained two sncRNAs that showed higher negative correlations between specific sncRNA abundance and putative target host gene expression. Among all detected sncRNAs, sncRNA_26 and 36 were detected in all four strains of BCTV. The highest abundance of sncRNA_26 was in the susceptible line (KDH19-17) and demonstrated a moderate negative correlation with the sugar beet target gene, UPF0554 protein C2orf43 homolog (*EL10Ac7g16816*), expression. The sncRNAs that were exclusive to the CA/Logan strain included sncRNA_4, sncRNA_20, sncRNA_21 targeting sugar beet leucine-rich repeat-containing protein 46 (*EL10Ac1g01206*), 7-deoxyloganetic acid glucosyltransferase (*EL10Ac5g12605*), and transmembrane emp24 domain-containing protein p24delta3 (*EL10Ac6g14074*) genes, respectively, and showed a strong negative correlation (−0.89 to −0.93) with the expression of the putative target genes. The sncRNA_16 was detected only in the Svr strain that showed moderate negative correlation (−0.54) with sugar beet pentatricopeptide repeat-containing protein (*EL10Ac4g08848*) gene expression (Table 2).

### 2.6. Validation of BCTV Strain Specific sncRNA Putative Target Sugar Beet Genes through Degradome Sequencing

The sncRNAs originating from different BCTV strains and their putative sugar beet gene targets were further validated using degradome sequencing (Table 3). The susceptible genotype, Line 19, was chosen for this work since, as at the early infection stage (6 dpi), significantly higher number of BCTV-sncRNA reads were observed in the ‘S’ line in comparison to fewer reads in the ‘R’ lines. Some sncRNAs were specific to a single BCTV strain (sncRNA_24: CA/Logan) and some were common to multiple BCTV strains (sncRNA_2: all 4 strains) and targeted multiple genes and/or multiple sequences of a specific transcript. The sncRNA_2 specific to Col and Svr strains, targeting *EL10Ac8g19233* (PRA1 family protein F3-like), showed higher degradome value (mean FPKM: 1412) in the infected samples vs. uninfected control (mean FPKM: 0). In another case, sncRNA_10 had multiple sugar beet gene targets showing higher degradome values in the infected samples for *Bevul.2G165300* (uncharacterized protein LOC104890896; mean FPKM: 362), *EL10Ac8g20333* (splicing factor 3B subunit 2; mean FPKM: 167), and *Bevul.9G034200* (uncharacterized protein LOC104890896; mean FPKM: 342) vs. uninfected control samples for the same genes (mean FPKM: 0). An elaborate list of all other sncRNAs and their putative sugar beet gene targets is shown in Table S7. Target plots (t-plots) of some of the highly significant (*p* < 0.05) sncRNAs (e.g., sncRNA_2) and/or sncRNAs with high degradome reads (e.g., sncRNA_1) are shown in Figure S6.

Gene ontology (GO) analysis of BCTV sncRNAs putative target sugar beet genes (based upon degradome data) at 6 dpi were primarily associated with the chloroplast, cytosol, ribosome, etc. (Figure S7A). Pathway enrichment analysis revealed genes predominantly associated with the ribosome, protein processing in the endoplasmic reticulum, glyoxylate and dicarboxylate metabolism, amino sugar and nucleotide sugar metabolism, etc. (Figure S7B).

### 2.7. BCTV Strain Specific Divergence in Relation to Functional Elements

**Figure 8:**
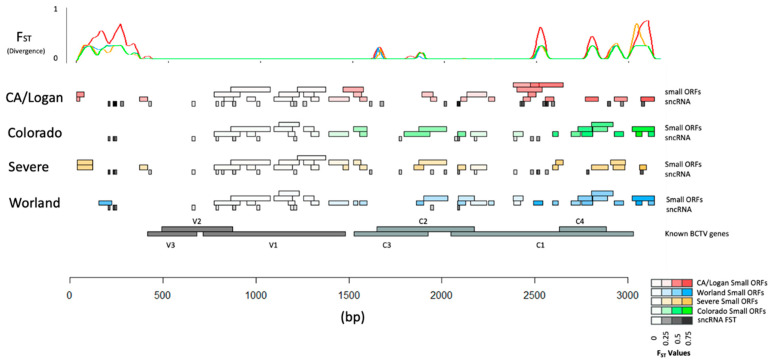
Divergence with respect to functional elements in the genomes of four Beet curly top virus (BCTV) strains. Higher values of FST at specific locations in the genomes are associated with greater divergence as evident by the peaks [small ORFs: small Open Reading Frames; small non-coding RNA: sncRNA; V3: movement protein; V2: SS-DS DNA regulator; V1: capsid protein; C3: replication enhancer; C2: pathogenesis enhancement protein; C1: rolling circle replication initiator protein; C4: cell cycle regulator].

Divergence of underlying sequence was estimated using pairwise F_ST_ between populations representing the four BCTV strains (CA/Logan, CO, Svr, and Wor). Divergence was calculated at each locus in the genome in a way that divergence could be viewed with respect to strain and functional elements (e.g., genes, sORFs, and sncRNA) (Figure 8; Tables S8–S10). Average divergence with respect to strain showed CA/Logan as the most diverged F_ST_ = 0.165. Average divergence of CO, Wor, and Svr were also estimated and F_ST_ was equal to 0.104, 0.112, and 0.126, respectively. The comparatively smaller values of divergence between CO and Wor may reflect their close relationship and treatment as unique groups. The variance within subpopulation was calculated, denoted by Hs, with values ranging from 0.020 to 0.033.

Specific regions with low divergence appeared to coincide with the BCTV genes V3 (product movement protein), V2 (product SS-DS DNA regulator), and V1 (product capsid protein). The low divergence either reflects high sequence similarities between strains or high variability with strains. Several genome-wide patterns emerged based on divergence between strain genomes and specific features could be identified. First, the non-coding region 0 to 300 bp and the coding region from 1500 bp to 3750 bp contained most of the strain specific variation as indicated by divergence. Perhaps, not coincidentally, the majority of strain specific sncRNA and ORFs were found in these regions. Estimates for strain specific divergence for sncRNA (Table S8), BCTV genes (Table S9), and ORF (Table S10) were performed. Divergence in C3 (replication enhancer), C2 (pathogenesis enhancement protein), C1 (rolling circle replication initiator protein), and C4 (cell cycle regulator) was much greater (Table S9) than for V1, V2, and V3.

### 2.8. Differential Regulation of sORF Derived Peptides Originating from the BCTV Strains

The susceptible line, KDH19-17, was used to investigate any putative sORFs derived peptides originating from the different BCTV strains at early and late infection stages. The different peptides identified in our study showed amino acid sequence identities with sORFs originating from all four BCTV strains. The CA/Logan strain derived peptides that were detected in the newly emerging leaves at early infection stage (6 dpi) corresponded to sORFs 1, 6, 7, 9, 10, 16, 17, 20, 22, and 15 (Table S11). Whereas, at later infection stage [1 month post inoculation (mpi)], peptides primarily corresponded to sORFs 1, 6, 16, 20, and 23 in addition to sORFs 2, 5, 7, 8, 9, 13, 15, 19, and 24. The CO strain derived peptides that were detected at 6 dpi corresponded to sORFs 4 and 15 (Table S12). At 1 mpi, peptides primarily corresponded to sORF 4 and 15 in addition to sORFs 1, 2, 4, 5, 8, 11, and 14. The Svr strain derived peptides detected at 6 dpi primarily corresponded to sORFs 2, 6, and 11 in addition to other detected peptides originating from sORFs 4, 14, and 17 (Table S13). At 1 mpi, peptides primarily corresponded to sORFs 2, 11, and 17 in addition to sORFs 6, 7, 10, 12, 15, and 16. The Wor strain derived peptides detected at 6 dpi primarily corresponded to sORFs 5, 7, and 13 in addition to other detected peptides originating from sORFs 16 and 19 (Table S14). At 1 mpi, peptides primarily corresponded to sORFs 5, 7, 13, 16, 17, and 19 in addition to sORFs 1, 4, 6, and 18.

## 3. Discussion

Host plant resistance mechanisms against viruses are multilayered and deployed at the level of transcription, post-transcriptional silencing, translation, and metabolites [10,17,31]. One of the primary goals of this study was to identify possible mechanisms at early stages of BCTV infection (with no visible disease symptoms) that contribute to resistance visible at later stages of BCTV infection. Multi omics tools were used to dissect putative disease resistance mechanisms in the ‘R’ lines during sugar beet–BCTV interactions. Global gene expression analysis showed induction of several candidate genes associated with diverse functional categories and their putative roles in sugar beet resistance. Examples of some of the differentially expressed genes in the ‘R’ lines whose expression were several folds higher (vs. ‘S’) at 2 dpi include *EL10Ac3g06016* (ribosome related), *EL10Ac5g11973* (protein FIZZY-RELATED 3-like isoform X5), *EL10Ac5g10437* (inhibitor of trypsin and hageman factor), *EL10Ac5g12445* (F-box protein), *EL10Ac6g14424* (MAPK signaling), and *EL10Ac7g16530* (polyubiquitin). At 6 dpi examples of candidate genes that were highly up-regulated in the ‘R’ lines (vs. ‘S’) include *EL10Ac6g14636* (jasmonate-induced protein), *EL10Ac2g02812* (probable prolyl 4-hydroxylase 10), *EL10Ac4g08162* (putative 1-phosphatidylinositol-3-phosphate 5-kinase FAB1D) (Figure 1; Tables S2 and S3). More than 60-fold upregulation (Table S2) of the gene, *EL10Ac5g10437* (inhibitor of trypsin and hageman factor), in the ‘R’ lines (vs. ‘S’) at 2 dpi and known function of this gene against insect pests [32] might possibly indicate sugar beet responses against the vector, BLH. Some of the highly up-regulated genes identified in this study are in line with their protective roles against viruses in other plant systems [1,10]. Higher basal (in absence of the virus) expression of genes such as *EL10Ac6g13092* (ras-related protein RAB1BV-like) and *EL10Ac3g06086* (70 kDa peptidyl-prolyl isomerase) in the ‘R’ lines might provide insights on putative roles of these genes in host defense priming against the virus. Phytohormones such as JA and SA are known to activate defense related pathways in plants in response to viruses [9,11] and also insect pests [33] suggesting overlap in functions. Significant up-regulation of JA related specific candidate genes in the ‘R’ lines at 2 dpi and 6 dpi observed in this study are in line with earlier observations on the role of JA/derivatives in plant viral resistance and may also be in response to the insect pest. Further analysis of JA/derivatives showed higher amounts, e.g., in Line 4 at 2 d both in the control and infected samples (vs. corresponding ‘S’ control and infected samples) (Table S6). Foliar application of Met-jasmonate in sugar beets reduced viral titers in the leaves and improved resistance against Beet mosaic virus [34] through modulation of SMs and a phytohormone (SA) known to confer resistance against different of biotic and abiotic stresses in plants [35]. Differentially expressed genes in the control plants (Figure S2) differed from the infected plants suggesting differential regulation post BCTV infection. As an example, some of the highly up-regulated genes in the ‘R’ lines (vs. ‘S’) were primarily associated with amino acid metabolism. Pathway enrichment analysis of DE genes indicated differential regulation of carbon and nitrogen metabolism and secondary metabolites during early stages of BCTV infection in the ‘R’ and ‘S’ lines (Figure 3) in contrast to the control plants where DE genes were associated with amino acids, glutathione, and flavonoid metabolism (Figure S4). Differentially expressed genes that showed greater fold changes in the ‘R’ lines but no known functions/uncharacterized (such as *EL10Ac9g20974* and *EL10Ac2g03119*), could be an interesting topic for future exploration to further understand their roles in BCTV resistance.

Weighted gene co-expression network analysis of DE genes, helped in narrowing down to key gene modules (MEblue, MEgrey60, and MEorange) and representing candidate genes (such as *EL10Ac3g07378*; brassinosteroid insensitive 1-associated receptor kinase, *EL10Ac8g19281*; eukaryotic membrane protein, *EL10Ac9g22911*; organic cation/carnitine transporter 2, *EL10Ac9g22672*; NAD-dependent protein deacetylase SRT1, *EL10Ac9g22628*; zinc finger CCCH domain-containing protein 20, *EL10Ac6g14628*; protein PHYLLO, chloroplastic) that could putatively be associated with resistance in the ‘R’ lines (Figure 4C, Table S16). Future validation of these candidate genes using genomic sequencing and expression analysis in a segregating population (exhibiting resistance and susceptibility) resulting from crosses between ‘S’ and ‘R’ lines (currently underway), will precisely define their roles in sugar beet BCTV resistance.

The untargeted metabolome analysis used in this study identified metabolites that could potentially be associated with resistance. The metabolites that were distinctly different and were in much higher contents in the leaves of ‘R’ lines (vs. ‘S’) include isoflavonoid O-glycosides, flavonoid 8-C glycosides, triterpenoid, and iridoid-O-glycosides to name a few (Figure 5 and Figure 6). Interestingly, all these metabolites were at much higher levels in the leaves of ‘R’ lines at the basal level, i.e., in absence of BCTV infection. Infection with the virus did not alter their levels in the ‘R’ lines suggesting that their basal levels might had been enough to provide necessary protection against the virus. The role of flavonoids, isoflavonoids, terpenoids, etc. in plant resistance against viruses and other pathogens are well documented [17]. Mechanisms through which these metabolites confer host plant resistance include modulating host plant cell signaling pathways, pathogen enzyme inhibition, DNA alkylation, and interfering with the reproduction system. Results from this study in sugar beet identifies specific flavonoid/isoflavonoid/terpenoid derivatives, such as vitexin 2″-glucoside and 9-methoxy-7-[4-[3 4 5-trihydroxy-6-[[3 4 5-trihydroxy-6-(hydroxymethyl)oxan-2-yl]oxymethyl]oxan-2-yl]oxyphenyl]-[1 3]dioxolo[4 5-g]chromen-8-one with putative protective roles against BCTV. Higher content of the triterpenoid, ursolic acid, in the leaves of ‘R’ lines (Figure 5 and Figure 6) might also indicate its protective role against BCTV. Antiviral properties of ursolic acid against both plant and animal related viruses [36] and antimicrobial properties of ursolic acid [37] have been reported in several studies. Foliar application of ursolic acid in tobacco (*Nicotiana benthamiana*) prior to infection with Tobacco mosaic virus (RNA virus) increased host plant resistance by significantly reducing viral titers and increasing activities of antioxidant related enzymes (superoxide dismutase and peroxidase) along with increased expression of regulatory and defense related genes associated with the SA signaling pathway [38]. The specific flavonoid/isoflavonoid/triterpenoid derivatives identified in this work can be purified from the ‘R’ lines in the future and tested for their potential antiviral activities against BCTV. This may provide future opportunities for foliar application using these compounds (depending upon their antiviral potencies) to improve BCTV resistance in sugar beets.

Molecular mechanisms which differentiate specific strains of BCTV interacting with sugar beet genotypes and contribute to the development of disease symptoms are not well understood. The genomes of BCTV strains are similar in regard to their overall genetic composition, yet F_ST_ showed moderate levels of divergence at specific regions associated with different strains (Figure 8). Strain specific features (e.g., sncRNA and sORF) might provide us deeper insight on their putative roles in pathogenicity. Both sncRNAs and ORFs sequence divergence in these regions might have implications on strain specific virulence on suitable hosts. Most notable in this analysis was the ability to identify features which have potential functional consequences. Variations in the genomes of BCTV strains may condition the virus to produce different sncRNAs and sORFs (small peptides) that are involved in pathogenicity and host specific responses in sugar beet genotypes as has been noted in other Geminiviruses recently [21,22]. In our earlier work we have described BCTV sncRNAs and their putative sugar beet target genes [20]. BCTV derived sncRNAs had multiple targets, but the best hits based on the abundance of sncRNA/s and their effect on sugar beet target gene expression were taken into consideration for analysis. The sncRNA_4 (GTTCAAAAGATTGTGATGTTGAAGG), 20 (GCTTCTTCTTTTGAAAG), and 21 (GAGATATGAACAAGAGG) detected in this study, and their effects on putative sugar beet target genes *EL10Ac1g01206* (*leucine-rich repeat protein*; LRR), *EL10Ac5g12605* (*7-deoxyloganetic acid glucosyltransferase*), and *EL10Ac6g14074* (*transmembrane emp24 domain protein*), respectively, are in accordance with our earlier report where a high negative correlation (~−0.90 and higher) was observed between their abundance and corresponding sugar beet target gene expression [20]. Here, we dissect BCTV strain specific sncRNAs and their putative sugar beet target genes using a combination of computational predictions (based on core sequence complementarity between sncRNA to sugar beet genes) and experimental data. Using sRNAseq and mRNAseq expression data derived from plants infected with BCTV strains, 37 different viral derived sncRNAs that shared sequence complementarity to target genes in the sugar beet genome were found. Out of the 37 sncRNAs, only 10 sncRNAs showed higher negative correlations between the abundance of a specific sncRNA and its target sugar beet gene expression that varied between susceptible vs. resistant genotypes. In some cases, sequence divergence of sncRNAs appeared to be correlated with a specific strain(s) and had potential functional consequences on target host gene expression (Table 2; Figure 8). As an example, sncRNA_36 was common to all four strains and showed moderate negative correlation with the expression of *EL10Ac9g22982.1* (transmembrane protein 53) gene in the susceptible genotype (Table 2). Whereas sncRNAs_4, 20, and 21 (highly abundant in the susceptible genotype) were observed only in the CA/Logan strain and showed high negative correlation with the expression of their corresponding target genes *EL10Ac1g01206* (*leucine-rich repeat protein*; LRR), *EL10Ac5g12605* (*7-deoxyloganetic acid glucosyltransferase*), and *EL10Ac6g14074* (*transmembrane emp24 domain protein*), respectively. The presence of sncRNA_26 and sncRNA_36 in all four strains suggests conservation of function across virus strains. Conversely, sncRNA_16, which targets the *EL10Ac4g08848* (*pentatricopeptide repeat-containing protein*; PPR) gene, was only detected in the *Svr* strain suggesting strain specificity (Table 2). Many of the BCTV target genes in sugar beet identified in this study have been shown to play important roles in resistance against diverse plant pathogens including viruses in other plants. Some examples include LRR and PPR genes. Several LRR genes have been identified and/or cloned to demonstrate their role in resistance against viruses [39]. PPR genes belong to large protein families in plants and are implicated in plant development and stress responses against a wide range of pathogens [40,41]. PPR genes are targeted to mitochondria and chloroplasts and modulate RNA properties. PPR’s RNA binding properties have been effectively used to genetically engineer plants conferring resistance against viruses [42]. In mammalian cells, suppression or deletion of a *transmembrane emp24* candidate gene, *TMED2*, increased the titer of herpes simplex virus 1 [43]. Not all detected BCTV derived sncRNAs altered expression of their corresponding sugar beet target genes at the early infection stage (6 dpi) when no visible disease symptoms were evident but may have implication at later stages of infection when disease symptoms are visible. On the other hand, plants can also overcome any inhibitory effects exerted by these sncRNAs.

Candidate genes identified based on high negative correlation resulting from high abundance of a specific BCTV sncRNA and low expression of a sugar beet target gene(s) (Table 2), differed from the targets identified through degradome analysis (Table 3 and Table S7). Significant degradation of sugar beet target transcripts by BCTV strain specific sncRNAs as evident from the degradome data (Table 3 and Table S7; Figure S6) necessarily did not alter the expression of target gene(s) (Table S7) at the early infection stage (6 dpi) suggesting possible increase in the rate of transcription vs. target transcript degradation. Degradome analysis at later stages of BCTV infection in the future could possibly explain the role of sncRNAs in degradation of target sugar beet transcripts. The two approaches used in this study to identify putative BCTV-sncRNA target sugar beet genes might indicate different mechanisms by which sncRNAs might target sugar beet genes that are critical in host plant defense against the virus. Virus derived sncRNAs are capable of methylating genes in the host plant genome and thereby could modulate their expression or sncRNAs can directly target host gene transcripts followed by cleavage at the target transcript site resulting in cross-kingdom RNAi [31]. A future whole-genome methylation study in control and infected sugar beet ‘S’ and ‘R’ lines at early and late infection stages will precisely determine the role of sncRNAs in DNA methylation and altering expression of sugar beet target genes (Table 2), if any.

The predicted sORFs and putative small peptides (Table S10) produced by them will be of interest for future studies as their specific roles as pathogenicity factors in BCTV strains is currently unknown. Recent studies have shed light on BCTV and related Geminiviruses that produce some of these small peptides with putative roles in virulence [21,22]. These roles were primarily proven through over-expression of selected sORFs that produce small peptides and/or mutation to understand their role in pathogenesis during plant infection. In our case, a peptidomics approach was used along with the BCTV susceptible genotype, KDH19-17, to identify any BCTV derived small peptides in sugar beet leaves during natural infection conditions. Our data show differential abundance of specific sORF derived peptides (Tables S11–S14) at early (6 dpi; no visible symptoms) and late (1 mpi; full blown symptoms) infection stages. As an example, a small peptide sequence ‘FECMCT’ originating from sORF1 in CA/Logan and sORF6 in Svr strains was detected only in CA/Logan and Svr strains (Tables S11 and S13) both at early and late infection stages. This may provide some insights on the role of these sORFs in pathogenesis and disease severity. In fact, both strains are known to produce strong BCTV symptoms in sugar beets [4]. Future functional characterization through deletion/mutation will determine their precise role in BCTV pathogenicity. These sORFs may also serve as potential targets for RNAi-based strategies for disease control in sugar beet. BCTV like other Geminiviruses have highly compact and efficient genomes. Though the peptide sequences aligned well with the sORFs, overlaps between the sORFs and main ORFs in some cases also raise the question that some of these detected peptides may have been originated from the main ORFs. The smaller size of the virus genome means that accomplishing fundamental functions such as viral replication, infection, and movement will require producing multifunctional proteins and sncRNAs that can be used to carry out these functions across a wide range of hosts and environments. The role of virus sncRNAs and sORFs taken together into consideration possibly explain the functional implication of BCTV genome diversity contributing to silencing of plant genes (through sncRNAs) involved in host defense and the production of putative viral virulence proteins (small peptides originating from sORFs) putatively associated with pathogenicity and disease severity.

## 4. Materials and Methods

### 4.1. Plant Growth Condition, Viral Infection of Sugar Beet Plants, and Sample Collection

BCTV susceptible (KDH19-17; Line 19) and resistant (KDH13; Line 13 and KDH4-9; Line 4) sugar beet plants at 4–5 leaf-stage were infected with viruliferous BLHs (~6–8 BLH/plant) primarily carrying (higher relative abundance) BCTV strains CA/Logan and Svr, but also carry other BCTV strains including CO and Wor which are prevalent in sugar beet growing regions in the western United States [6,20]. Plants were put inside cages containing viruliferous hoppers and exposed for 2 d and 6 d. The cages were maintained in a growth chamber with the following conditions: 28 °C (day)/21 °C (night), 16 h (day)/8 h (night) photoperiod, and 20% relative humidity. Uninfected plants under the same growth chamber conditions but in separate cages served as controls. Following 2 days post inoculation (dpi) and 6 dpi with BLHs, leaf samples (2–3 apical leaves/plant) designated as early timepoint samples from both infected (5 biological replicates; each replicate consisted of leaf tissues obtained from a single plant) and uninfected plants (3 biological replicates; each replicate consisted of leaf tissues obtained from a single plant) were collected, flash frozen in liquid N, and stored at −80 °C until further processing. Post sample collections at 2 dpi and 6 dpi, the infected plants were sprayed with Admire^®^ Pro (Bayer CropScience LLC, Research Triangle Park, NC, USA) insecticide to eliminate any existing BLHs and future infections. The uninfected plants were also treated the same way with Admire^®^ Pro, like the infected ones. The plants were then sprayed with water to remove any insecticide residues and moved to the greenhouse and evaluated for BCTV symptoms such as leaf vein swelling, leaf thickening, and curling predominantly in the apical leaves [20] (Figure S8) at 3-week post inoculation (wpi) along with uninfected control plants that did not show any disease symptoms. The removal of apical leaves had minimal effects on improving disease symptoms and/or viral titers [20] as full blown BCTV symptoms were evident in the ‘S’ line (vs. ‘R’ lines). Apical leaves collected from the infected (6 d exposure to BCTV) ‘S’ line plants showing BCTV symptoms at 1 month post inoculation (1 mpi), were designated as later infection stage/late time point samples. Three biological replicates for each of the early and late time points infected samples (each replicate contains leaf tissues pooled from 2 plants), were similarly flash frozen in liquid N and stored at −80 °C until further processing for viral peptidome related work. A schematic diagram of the experimental design is presented in Figure S9.

### 4.2. Extraction of Total RNA, sRNA and mRNA Library Preparations, and Sequencing

The ‘Plant/Fungi Total RNA Purification Kit’ (Norgen Biotek Corp, ON, Canada) was used to isolate total RNA as per the manufacturer’s protocol. RNA quality and quantity were analyzed using Bioanalyzer 2100 (Agilent Technologies, Santa Clara, CA, USA). RNA samples with a RIN number > 7.0 were used to prepare small RNA sequencing (sRNAseq) and messenger RNA sequencing (mRNAseq) libraries. For both sRNAseq and mRNAseq libraries, approximately 1 ug of total RNA were used. Illumina Hiseq 2500 sequencing platform and 50 bp single-end sequencing approach was used at LC Sciences (Houston, TX, USA) according to the vendor’s protocol [20]. For mRNAseq libraries, ribosomal RNA depletion was performed following the protocol described in the Ribo-Zero™ rRNA Removal Kit (Illumina, San Diego, CA, USA). Oligo-(dT) magnetic beads were used to purify poly(A) mRNA. Using a divalent cation buffer under elevated temperature, poly(A) RNA was fragmented. This procedure was followed by reverse-transcription of cleaved mRNA fragments to produce cDNA that was subsequently used to produce U-labeled second strand DNA. Following end repair, 3′ adenylation, adapter ligation, and PCR, final libraries were made. Bioanalyzer 2100 was used for quantification and quality control of the mRNAseq libraries. Illumina’s NovaSeq 6000 sequencing platform was used to perform paired-end (150 bp) sequencing.

### 4.3. Read Mapping and Transcriptome Assembly

mRNA-Seq raw reads were processed using in house (LC Sciences) Perl scripts and Cutadapt (1.10) [44]. Low-quality reads and adapter sequences were removed followed by FastQC (0.10.1) (http://www.bioinformatics.babraham.ac.uk/projects/fastqc/, accessed on 15 April 2021) to evaluate sequence quality. Using Hisat (2.0) [45], the reads were mapped to the EL10.1 (https://phytozome-next.jgi.doe.gov/, accessed on 15 April 2021) sugar beet (*Beta vulgaris* subsp. *vulgaris*) reference genome. The mapped reads from each sample were assembled through StringTie (1.3.4) [46].

### 4.4. Differential Expression of mRNAs and Bioinformatics Analysis

Transcriptome data that originated from 46 different samples [20] were merged to reconstruct comprehensive transcriptome data through Perl scripts. StringTie (1.3.4) [46] and edgeR (3.42) [47] were used to quantify expression levels of transcripts and expressed as FPKM (fragments per kilobase of transcript per million mapped reads). Differential expression was performed using EdgeR-R packages. A *p*-value of <0.05 and |log2 (fold-change)| ≥ 2 or ≤−2 were used to identify differentially expressed genes (DEGs). Blastx was used for annotation of transcripts against the NCBI database. The ‘R’ lines (Line 13 and 4) were compared to the ‘S’ line (Line 19) both at 2 d and 6 d within uninfected control (C) and BCTV infected (I) treatments. The Venn diagrams generated for a specific time point (2d/6d) and treatment type (C/I) resulted from multiple comparisons involving comparison of each of the ‘R’ lines to the ‘S’ line.

Annotation of transcripts were performed using Blastx against the NCBI database. For Gene Ontology (GO) analysis, transcripts were blasted to the GO database to calculate the gene numbers for each term. Pathway enrichment was performed using the Kyoto Encyclopedia of Genes and Genomes (KEGG) [48].

The weighted gene co-expression network analysis (WGCNA) package in ‘R’ (3.2.2.) was used to perform WGCNA analysis using the methods described by the authors [49]. The parameters used for analysis included minimum module size (for module detection): 30; minCoreKME: a number between 0–1. If a detected module does not have at least minModuleKMESize genes with eigengene connectivity at least minCoreKME, the module is disbanded (its genes are unlabeled and returned to the pool of genes waiting for module detection): 0.5; and min CoreKMESize (see minCoreKME): minModuleSize/3; minKMEtoStay (genes whose eigengene connectivity to their module eigengene is lower than minKMEtoStay are removed from the module): 0.3. For each block of genes, the network is constructed, and topological overlap is calculated. The topological overlaps are returned as part of the return value list. An average linkage hierarchical clustering method is used for clustering genes followed by the identification of modules in the resulting dendrogram by the Dynamic Hybrid tree cut. Found modules are trimmed of genes whose correlation with module eigengene (KME) is less than minKMEtoStay. Modules in which fewer than minCoreKMESize genes have KME higher than minCoreKME, are disbanded.

### 4.5. Analysis of sncRNAs Derived from the BCTV Genomes

Sequence reads originating from the sRNAseq data were aligned to a representative genome for each BCTV strains: CA/Logan CTS06-104 (GenBank accession KX867032.1), CO CTS15-113 (KX867056.1), Svr CTS06-021 (KX867019.1), and Wor CTS15-095 (KX867055.1). Reads were aligned using BWA-MEM [50]. The ‘.sam’ files resulting from this analysis were converted to ‘.bam’ files and sorted through Samtools v1.9 [51]. Reads showing high quality alignments (Q > 30) to the BCTV genomes were used for further analysis. The converted (.fasta) reads were then reverse completed using a python script (https://www.bioinformatics.org/sms/rev_comp.html, accessed on 29 March 2022). These sequences were then used as queries to find host targets based on homology. A BLAST analysis was performed against the sugar beet EL10.1 cDNA sequences using the blastn [52] program. Core sequence homology of sncRNAs (31–41 nucleotide long) were those small sncRNAs that aligned to the virus and the reverse complement of which had an 18–21 nucleotide sequence, that had a perfect (100%) or near perfect match to the sugar beet host target gene(s) in the genome of EL10.1. We used size of match (18–21 nt), and percent identity to choose sncRNA targets. The expression of sncRNA target genes in sugar beet were evaluated by examining their FPKM values between treatments. Correlations between a specific sncRNA abundance and corresponding sugar beet target gene(s) expression (FPKM) was performed using ‘R’ (version 4.2.1). In this way putative targets within the virus and host genomes could be identified.

### 4.6. Visualization of Transcription in the Virus Genomes

The ‘R’ (version 4.2.1) software package was used to visualize the virus genome. The gene positions in the virus genomes were extracted from the CA/Logan, CO, Svr, and Wor strains. The coverage of RNAseq reads across the BCTV genomes was determined using Samtools depth. Read depth were plotted across the BCTV genomes covering main ORFs, sORFs, and regions producing sncRNAs. Data extracted from BCTV genomes were transformed into circular plots using functions as described in the ‘Rcircos’ source code [53].

### 4.7. Data and Code Availability

The sRNAseq (BioProject ID: PRJNA764694) and mRNAseq (BioProject ID: PRJNA764690) raw data are available at the NCBI SRA database. The codes used are available at https://github.com/BetaGenomeNinja/BCTV_sncRNA (accessed on 24 September 2023). Unique sugar beet targets and expression of host genes with respect to genotype were determined previously with the exception that the sncRNA could be partitioned into strain differences as the result of preferential alignment [20]. ORF detection for the four strains was carried out using ORFfinder (https://www.ncbi.-nlm.nih.gov/orffinder/, accessed on 3 February 2022), setting the minimum length to 30 nt and limiting the start codon to ATG only. The sequences for specific BCTV genes were derived from NCBI for each of the representative virus strains: CA/Logan, CO, Svr, and Wor.

### 4.8. Population Genomic Analysis

A single consensus sequence representing each of the four BCTV strains was used to determine physical position and orientation of features (e.g., sncRNA, ORFs, proteins, and genes) using blastn. This allowed the comparison of features and underlying sequences. Population genetic methods were used to determine variance within and between strains and to calculate divergence (F_ST_).

HS = Variation within sub-population (1 − Σps^2^)

HT = Variation within all populations (1 − Σpt^2^)

ps = Allele frequency (p) in subpopulation (s)

pt = Allele frequency (p) in total (t)

$$FST=\frac{HT-HS}{HT}$$

We reported HS and F_ST_ to get a sense of the variation within and between strains. Divergence (F_ST_) was used to identify the specific variation underlying these relationships relative to the functional regions within the BCTV genome.

Low HS and low divergence represent conservation of sequence and sequence similarity. Low HS and high F_ST_ represent divergence and unique variation with respect to strain or cluster. High HS generally represented low divergence. Yet some sites were high HS and moderate divergence levels suggesting a divergence between clusters within a strain.

### 4.9. Degradome Library Construction and Sequencing

Total RNA was extracted using ‘Plant/Fungi Total RNA Purification Kit’ (Norgen Biotek Corp, Thorold, ON, Canada) following the manufacturer’s protocol. RNA quality and quantity were analyzed using Bioanalyzer 2100 and RNA 6000 Nano Lab Chip Kit (Agilent, Santa Clara, CA, USA) with a RIN number > 7.0. Approximately 20 ug of total RNA were used to prepare Degradome library. Approximately 150 ng of poly(A) + RNA was used as input RNA. This was followed by annealing RNAs to Biotinylated Random Primers and streptavidin capture of RNA fragments bound to Biotinylated Random Primers. This was followed by 5′ adaptor ligation to RNAs containing 5′-monophosphates, reverse transcription, and PCR amplification. Resultant libraries were then sequenced using the 5′ adapter sequences only, sequencing the first 36 nucleotides of the inserts that represented the 5′ ends of the original RNAs. This was followed by single-end 50 bp sequencing on an Illumina Hiseq 2500 platform (LC Sciences, Houston, TX, USA) following protocol recommended by the vendor.

### 4.10. Degradome Data Analysis

Degradome data (PRJNA1009842; submitted to the NCBI SRA database) were analyzed using the ACGT101-DGD-v4.0 pipeline (LC Sciences, Houston, TX, USA). Raw sequencing reads were processed using Cutadapt [44] and in-house perl scripts were used to remove adaptors and low-quality reads. The processed sequencing reads were then used to identify potentially cleaved targets through the CleaveLand pipeline [54]. The degradome reads were mapped to the mRNA downloaded from the sugar beet genome database (https://phytozome-next.jgi.doe.gov/, accessed on 26 February 2023). Only the perfect matching alignment(s) for given reads were kept for degradation analysis. All resulting reads (t-signature) were reverse complemented and aligned to the BCTV sncRNAs identified in our study. Alignments where the degradome sequence position coincided with the tenth or eleventh nucleotide of sncRNA were retained and scored. The targets were selected and categorized as 0, 1, 2, 3, or 4. Category 0: over one raw read at the position, abundance at the position is equal to the maximum on the transcript, and there is only one maximum on the transcript; category 1: over one raw read at the position, abundance at the position is equal to the maximum on the transcript, and there is more than one maximum positions on the transcript; category 2: over one raw read at the position, abundance at position is less than the maximum but higher than the median for the transcript; category 3: over one raw read at the position, abundance at the position is equal to or less than the median for the transcript; and category 4: only one raw read at the position. In addition, to analyze the sncRNA targets and RNA degradation patterns, t-plots were constructed according to the distribution of signatures (and abundances) along these transcripts. All the identified targets were subjected to BlastX analysis to search for similarities and followed by GO analysis to uncover the sncRNA-gene regulatory network based on biological process, cellular component, and molecular function. Significant KEGG pathways were calculated as per the hypergeometric equation shown below. TB gene number = number of total genes; TS gene number = number of target genes in total genes; B gene number = total number of genes in KEGG pathways; S gene number = number of target genes in this KEGG pathway. Those KEGG pathways with *p* < 0.05 were defined as significant KEGG pathways.

$$P=1-\sum_{i=0}^{S-1}\frac{\left(\begin{array}{l} B\\ i \end{array}\right)\left(\begin{array}{l} TB-B\\ TS-i \end{array}\right)}{\left(\begin{array}{l} TB\\ TS \end{array}\right)}$$

### 4.11. Untargeted Metabolomics: Sample Preparation, Running, and Analysis

Methanol, acetonitrile, and LC/MS grade formic acid and water were purchased from Fisher Scientific. Sugar beet metabolites were extracted from leaf samples (100 mg) with 1 mL methanol for 16 h in the dark on an orbital shaker (5000 rpm). The extracts were centrifuged (16,800× *g*, 6 min) to pellet the extracted leaf material. The supernatants were transferred to a clean tube and concentrated via speedvac (Savant, Thermo Fisher Scientific, Waltham, MA, USA). Each extract was re-dissolved in methanol (250 µL) and any particulates were removed via centrifuge (16,800× *g*, 2 min).

The re-dissolved, centrifuged extracts were analyzed on a Waters Acquity ultra-performance liquid chromatography (UPLC) system coupled to a Waters Xevo G2 XS Quadrupole Time-of-Flight (QTOF) mass spectrometer (MS). The QTOF MS was equipped with a Z-spray ionization source running in ESI+ mode using MassLynx 4.2 software with the following settings: source temperature: 100 °C, desolvation temperature: 250 °C, desolvation gas flow: 600 L/h, cone gas flow: 50 L/h, capillary voltage: 3.0 kV, and sampling cone voltage: 40 V. Analyses were performed in sensitivity and continuum mode, with a mass range of *m*/*z* 50–1200 and a scan time of 0.1 s. A data-independent acquisition (DIA) method with elevated collision energy (MS^E^) was used with 6 eV low energy and a high energy ramp from 10−45 eV. Injection volume was 1 µL. Separation was performed on a Waters BEH C18 1.7 µm, 2.1 × 50 mm column with the following gradient solvent system: (0.5 mL/min, solvent A: 0.1% formic acid in water; solvent B: 0.1% formic acid in acetonitrile: 5% B (0.00–1.25 min.), gradient to 25% B (1.25–1.50 min.), gradient to 100% B (1.50–5.00 min.), 100% B (5.00–7.50 min.), then column equilibration (5% B, 7.60–10.00 min.). Data were imported into MS-DIAL 5.1 [30] after file conversion using Analysis base file (Abf) converter (Reifycs, Tokyo, Japan). Compound identification was accomplished with MS-DIAL by matching MS/MS fragmentation from the MS-DIAL database consisting of ESI(+)-MS/MS from authentic standards (16,481 unique compounds).

### 4.12. Sample Preparation and Running for Peptidomics Analysis of BCTV Derived Small Peptides

Peptidomics analysis was performed according to the vendor’s (Creative Proteomics, Shirley, NY, USA) protocol [55]. Approximately 150 mg of previously frozen ground leaf tissues (from infected sugar beet plants) collected at 6 dpi and 1 mpi and stored in −80 °C were used for protein extraction using 0.1% trifluoroacetic acid (TFA) as a lysis buffer. Following the addition of the lysis buffer (1 mL), the samples were incubated at 4 °C for 20 min with shaking every 5 min. This was followed by centrifugation at 14,000 rpm for 30 min at 4 °C and transferring the supernatant into new tubes. This was followed by transferring the solution into Microcon devices YM-10 (MilliporeSigma, Burlington, MA, USA) and centrifugation at 12,000 rpm at 4 °C for 10 min (repeated thrice). The filtrate was collected in the ultrafiltration tube.

Activation of the ziptip column was performed using 100% acetonitrile followed by equilibration with 0.1% TFA. Samples were dissolved in 0.1%TFA and loaded on the ziptip column. Desalting the ziptip column was accomplished using 0.1% TFA followed by elution with 60% acetonitrile twice. All these steps, starting from activation, were performed twice.

The samples were run on a 3000 nano Ultra-High-Performance Liquid Chromatography (UHPLC) system (Thermo Fisher Scientific, Waltham, MA, USA) that was coupled with a Obitrap Q Exaxtive HF (Thermo Fisher Scientific, Waltham, MA, USA) mass spectrometry (MS) system.

The UHPLC system was attached to a trapping column (PepMap C18, 100Å, 100 μm × 2 cm, 5μm) and an analytical column (PepMap C18, 100Å, 75 μm × 50 cm, 2 μm). The loaded sample amount was 1 μg. The mobile phase consisted of A: 0.1% formic acid in water and B: 0.1% formic acid in acetonitrile and the total flow rate was adjusted to 250 nL/min. Linear gradient settings were from 2 to 8% buffer B in 5 min, from 8 to 20% buffer B in 60 min, from 20 to 40% buffer B in 33 min, then from 40 to 90% buffer B in 4 min.

The MS protocol included a full scan ranging between 300–1650 *m*/*z* at the resolution of 60,000 at 200 *m*/*z*. The automatic gain control target for the full scan was set to 3E6. The MS/MS scan was operated in Top 20 mode using the following settings: resolution 15,000 at 200 *m*/*z*; automatic gain control target 1E5; maximum injection time 19 ms; normalized collision energy at 28%; isolation window of 1.4 Th; charge sate exclusion: unassigned, 1, >6; and dynamic exclusion 30 s.

The raw files were analyzed using the PEAKS^®^ Studio 8.5 software. The parameters were set as follows: the protein modifications were none; the enzyme specificity was set to none; the precursor ion mass tolerance was set to 20 ppm, and MS/MS tolerance was 0.5 Da. Thus, identified peptides with high confidence were chosen for downstream protein identification analysis.

### 4.13. Bioinformatics Analysis of BCTV sORF Derived Peptides

Peptide sequences were aligned to each of the BCTV strain genomes using the program tblastn. Small peptides were evaluated based on the alignment position relative to predicted sORFs for each strain. Detection of peptides across replications and treatments also served as a means to reduce noise and determine the most significant peptides with respect to strain and time after infection.

## 5. Conclusions

This work demonstrated putative sugar beet resistance mechanisms against BCTV and possibly against the vector, BLH. Various omics tools were used to identify sugar beet genes (such as *EL10Ac5g10437* (inhibitor of trypsin and hageman factor), *EL10Ac6g14636* (jasmonate-induced protein), and *EL10Ac4g08162* (putative 1-phosphatidylinositol-3-phosphate 5-kinase FAB1D)) and metabolites (such as flavonoid and isoflavonoid glycosides) that are potentially involved in host plant resistance. Computational prediction combined with experimental data demonstrated that different BCTV strains produce critical heritable changes that might give rise to unique functionality related to pathogenicity. Degradome analysis identified potential BCTV-sncRNA(s) target genes (such as *EL10Ac8g19233* (PRA1 family protein F3-like) and *EL10Ac8g20333* (splicing factor 3B subunit 2)) in sugar beet. In addition, the sORFs derived peptides common in CA/Logan and Svr strains might indicate their putative roles during early and late pathogenesis and will be suitable candidates for further functional characterization in the future. The knowledge gained from this study and the experimental approaches used can be extended to other crop species that are susceptible to BCTV/Geminiviruses and/or other viruses. The information presented here will be useful in designing future mitigation strategies by controlling the expression of key genes and metabolites conferring sugar beet resistance against BCTV and/or suppressing transcription of critical viral pathogenicity factors using cutting-edge functional genomics tools.

## Figures and Tables

**Table 1:** Identification of small non-coding RNAs (sncRNAs) in the leaves of Beet curly top virus (BCTV) susceptible Line 19 at 6 days post inoculation (6 dpi), containing core motifs with sequence complementarity to putative sugar beet gene targets and originating from different BCTV strains known to infect sugar beets and produce disease symptoms [‘x’ sign denotes presence of specific sncRNA].

BCTV sncRNA	Sequence	Sugar Beet Gene ID	Gene Name	CA/Logan	Colorado	Severe	Worland
sncRNA_1	CTGGAGGAGGAAGAAAA	EL10Ac1g00033	Nitrate reductase [NADH]		x	x	
sncRNA_2	GTGGCCGAAGAAGAGGA	EL10Ac1g00783	Homeobox-leucine zipper protein HAT3	x	x	x	x
sncRNA_3	GCTTCATTTTCTGAGTTA	EL10Ac1g01113	Protein TRANSPARENT TESTA 12		x		
sncRNA_4	GTTCAAAAGATTGTGATGTTGAAGG	EL10Ac1g01206	Leucine-rich repeat-containing protein 46	x			
sncRNA_5	TATCAACCCCAAAATAT	EL10Ac1g02347	AP2-like ethylene-responsive transcription factor ANT	x			
sncRNA_6	GGGCTCTCTTCAAATCCCC	EL10Ac2g02425	Pentatricopeptide repeat-containing protein			x	
sncRNA_7	TTTCGGAGGAGGAAGAAAAA	EL10Ac2g02734	Cytosolic sulfotransferase 15			x	
sncRNA_8	GAAGAAGCTAGTGAGGT	EL10Ac2g04408	Kanadaptin	x		x	
sncRNA_9	CTTCAATATTTGAAGTA	EL10Ac2g04434	Auxin transport protein BIG	x			
sncRNA_10	ATCACTTTAAGTTTTTA	EL10Ac2g04915	Staphylococcal-like nuclease CAN1		x	x	
sncRNA_11	AAAGAAGAAAGAGGAAA	EL10Ac3g06583	Zinc finger CCCH domain-containing protein 32		x	x	
sncRNA_12	TTTTTCAAGAAATTGTT	EL10Ac3g06769	Pentatricopeptide repeat-containing protein	x			
sncRNA_13	CCCAAAATATGCATCAT	EL10Ac3g07263	Putative SWI/SNF-related matrix-associated actin-dependent chromatin regulator	x			
sncRNA_14	GTTGTGGTTGAATCTTT	EL10Ac3g07325	Putative disease resistance protein RGA3		x	x	
sncRNA_15	TGTAGCTCTCTGGCATT	EL10Ac4g08785	Heat shock 70 kDa protein 16	x			
sncRNA_16	TGCAGTGGAATTGTTTG	EL10Ac4g08848	Pentatricopeptide repeat-containing protein At1g11290			x	
sncRNA_17	TAATGATGAATTGTGAAA	EL10Ac4g09996	Aspartic proteinase-like protein 2			x	x
sncRNA_18	AAGGAAGTGAAGAAGCT	EL10Ac4g10022	Domain of unknown function (DUF3411)	x	x	x	x
sncRNA_19	AAGTGGGCCCCACAGGAA	EL10Ac5g10458	Hexose carrier protein HEX6	x		x	
sncRNA_20	GCTTCTTCTTTTGAAAG	EL10Ac5g12605	7-deoxyloganetic acid glucosyltransferase	x			
sncRNA_21	GAGATATGAACAAGAGG	EL10Ac6g14074	Transmembrane emp24 domain-containing protein p24delta3	x			
sncRNA_22	CATTTGAAGTTTGATAT	EL10Ac6g14625	DNA-directed RNA polymerase subunit beta	x			x
sncRNA_23	CATTTGAAGTTTGATATA	EL10Ac6g14832	Myosin heavy chain kinase B	x	x	x	x
sncRNA_24	GATGTTGAAGGAAGTAA	EL10Ac6g15173	(R,S)-reticuline 7-O-methyltransferase	x			
sncRNA_25	AATATTGAGGAAGTCTT	EL10Ac6g15406	Putative pentatricopeptide repeat-containing protein			x	
sncRNA_26	AGGTTTATTGTGAAGAA	EL10Ac7g16816	UPF0554 protein C2orf43 homolog	x	x	x	x
sncRNA_27	TGTCTGTTTACCTCCTC	EL10Ac7g16868	Casparian strip membrane protein 2	x			
sncRNA_28	ATTATACTATTATATCT	EL10Ac7g17297	Hypothetical	x	x	x	x
sncRNA_29	AAGGATATGGAGGGAAGGAGA	EL10Ac7g17983	Ras-related protein RABA5e	x		x	
sncRNA_30	AGAGGACTTGTGAGAGC	EL10Ac7g18186	Exocyst complex component EXO70A1		x	x	
sncRNA_31	ATATTAACATATCTATT	EL10Ac8g18763	Heparanase-like protein 3	x			
sncRNA_32	TTTTTCAAGACTTTCAAAAA	EL10Ac8g19534	Domain of unknown function (DUF4216)	x		x	
sncRNA_33	TTGAGGAAATACCAATT	EL10Ac9g21413	MADS-box protein AGL24	x			
sncRNA_34	AACTTTACTTTATTTAA	EL10Ac9g21740	Protein RAFTIN 1A	x			
sncRNA_35	ATGATGATATGTTGGGT	EL10Ac9g22691	Plant transposase	x		x	
sncRNA_36	AATGAAAGAAAAGAAAG	EL10Ac9g22982	Transmembrane protein 53	x	x	x	x
sncRNA_37	CATTACCACCTTTAATGA	EL10As5g23617	Putative pectinesterase inhibitor 45	x	x	x	x

**Table 2:** The most significant negative correlations between Beet curly top virus (BCTV) strain specific small non-coding RNA (sncRNA) abundance and corresponding sugar beet putative target gene expression at 6 days post inoculation (6 dpi). The numbers under each genotype denote reads of sncRNAs detected.

BCTV Strain	BCTV sncRNA	Sequence	Sugar Beet Gene ID	Gene Name	sncRNA Abundance per Sample			Target Gene Expression (FPKM)			Correlation
					KDH13 (R)	KDH19-17 (S)	KDH4-9 (R)	KDH13 (R)	KDH19-17 (S)	KDH4-9 (R)	
CA/Logan	sncRNA_4	GTTCAAAAGATTGTGATGTTGAAGG	EL10Ac1g01206	Leucine-rich repeat-containing protein 46	5	162	3	30.37	24.22	34.26	−0.93
CA/Logan	sncRNA_20	GCTTCTTCTTTTGAAAG	EL10Ac5g12605	7-deoxyloganetic acid glucosyltransferase	0	23	0	2.46	1.13	3.33	−0.92
CA/Logan	sncRNA_21	GAGATATGAACAAGAGG	EL10Ac6g14074	Transmembrane emp24 domain-containing protein p24delta3	1	35	1	14.65	12.75	16.20	−0.89
CA/Logan	sncRNA_36	AATGAAAGAAAAGAAAG	EL10Ac9g22982	Transmembrane protein 53	0	1	0	1.46	1.41	1.71	−0.63
CA/Logan	sncRNA_26	AGGTTTATTGTGAAGAA	EL10Ac7g16816	UPF0554 protein C2orf43 homolog	14	449	4	7.44	4.80	4.64	−0.44
Colorado	sncRNA_3	GCTTCATTTTCTGAGTTA	EL10Ac1g01113	Protein TRANSPARENT TESTA 12	0	1	0	5.98	3.72	6.58	−0.98
Colorado	sncRNA_10	ATCACTTTAAGTTTTTA	EL10Ac2g04915	Staphylococcal-like nuclease CAN1	0	1	0	8.54	6.10	7.76	−0.95
Colorado	sncRNA_26	AGGTTTATTGTGAAGAA	EL10Ac7g16816	UPF0554 protein C2orf43 homolog	0	36	0	7.44	4.80	4.64	−0.46
Colorado	sncRNA_30	AGAGGACTTGTGAGAGC	EL10Ac7g18186	Exocyst complex component EXO70A1	0	1	0	0.14	0.11	0.19	−0.78
Colorado	sncRNA_36	AATGAAAGAAAAGAAAG	EL10Ac9g22982	Transmembrane protein 53	0	1	0	1.46	1.41	1.71	−0.63
Severe	sncRNA_25	AATATTGAGGAAGTCTT	EL10Ac6g15406	Putative pentatricopeptide repeat-containing protein	0	1	0	3.41	3.03	3.20	−0.83
Severe	sncRNA_26	AGGTTTATTGTGAAGAA	EL10Ac7g16816	UPF0554 protein C2orf43 homolog	14	449	4	7.44	4.80	4.64	−0.44
Severe	sncRNA_30	AGAGGACTTGTGAGAGC	EL10Ac7g18186	Exocyst complex component EXO70A1	0	1	0	0.14	0.11	0.19	−0.78
Severe	sncRNA_36	AATGAAAGAAAAGAAAG	EL10Ac9g22982	Transmembrane protein 53	0	1	0	1.46	1.41	1.71	−0.64
Severe	sncRNA_16	TGCAGTGGAATTGTTTG	EL10Ac4g08848	Pentatricopeptide repeat-containing protein	0	4	0	10.97	8.59	8.73	−0.54
Worland	sncRNA_26	AGGTTTATTGTGAAGAA	EL10Ac7g16816	UPF0554 protein C2orf43 homolog	0	53	0	7.44	4.80	4.64	−0.46
Worland	sncRNA_36	AATGAAAGAAAAGAAAG	EL10Ac9g22982	Transmembrane protein 53	0	1	0	1.46	1.41	1.71	−0.63

**Table 3:** Beet curly top virus (BCTV) strain specific small non-coding RNAs (sncRNAs) and their putative target sugar beet genes at 6 days post inoculation (6 dpi) in the susceptible Line 19 obtained through degradome sequencing [I: infected; C: uninfected control; sncRNAs where *p* < 0.05 are in bold; ‘&’: cleavage site].

BCTV sncRNA	BCTV Strain	Target Sugar Beet Gene ID	Description	Target Transcript Sequence (5′-3′)	Degradome Reads		
					I (Mean FPKM)	C (Mean FPKM)	*p*-Value
sncRNA_1	Colorado, Severe	EL10Ac5g11423	aspartic protease in guard cell 1	CCUACUUCCUCUUCCAC & CUGGAGGAGGAAGAAAA	1044.7	0	0.10
**sncRNA_2**	CA/Logan, Colorado, Severe, Worland	EL10Ac8g19233	PRA1 family protein F3-like	UCUCUCUUCUUUGGCACC & GUGGCCGAAGAAGAG-GA	1411.5	0	**0.00**
sncRNA_9	CA/Logan	EL10Ac9g21455	snakin-2	ACUUCUCUCUCUUCUUG & AAAGAAGAAAGAGGAAA	547.0	0	0.10
**sncRNA_10**	Colorado, Severe	Bevul.2G165300	uncharacterized protein LOC104890896	UAAGAGCUUAAGG-GAC & AUCACUUUAAGUUUUUA	362.3	0	**0.02**
sncRNA_10	Colorado, Severe	EL10Ac2g04382	transport protein SEC31 homolog B isoform X2	AGGGAACUUAAAGAGAA & AUCACUUUAAGUUUUUA	137.7	0	0.07
**sncRNA_10**	Colorado, Severe	EL10Ac8g20333	splicing factor 3B subunit 2	AAAGAAUUUGAGGUGAA & AUCACUUUAAGUUUUUA	167.1	0	**0.04**
**sncRNA_10**	Colorado, Severe	Bevul.9G034200	uncharacterized protein LOC104890896	UAAGAGCUUAAGG-GAC & AUCACUUUAAGUUUUUA	341.9	0	**0.03**
sncRNA_11	Colorado, Severe	EL10Ac9g21455	snakin-2	ACUUCUCUCUCUUCUUG & AAAGAAGAAAGAGGAAA	523.2	0	0.10
sncRNA_16	Severe	EL10Ac6g13544	photosynthetic NDH subunit of lumenal location 5, chloroplastic	UAGUAAAUUCCGCUGCU & UGCAGUGGAAUUGUUUG	491.8	0	0.10
sncRNA_20	CA/Logan	EL10Ac4g08680	ACT domain-containing protein ACR12	CUUGCAAGGGGAGAAGC & GCUUCUUCUUUUGAAAG	142.3	0	0.07
sncRNA_20	CA/Logan	EL10Ac5g11755	5-methyltetrahydropteroyltriglutamate--homocysteine methyltransferase	UGGUCAAAAGGAUGAGGC & GCUUC-UUCUUUUGAAAG	212.4	0	0.10
sncRNA_24	CA/Logan	EL10Ac1g02173	E3 ubiquitin-protein ligase CIP8	CGACUUCC-UCCGCAUC & GAUGUUGAAGGAAGUAA	372.3	0	0.07
sncRNA_28	CA/Logan, Colorado, Severe, Worland	EL10Ac3g06671	28 kDa ribonucleoprotein, chloroplastic	CAAUAUGGUAGUGUGGU & AUUAUACUAUUAUAUCU	544.5	0	0.07
sncRNA_34	CA/Logan	EL10Ac1g01692	pre-mRNA-processing protein 40A isoform X1	UUGAAGAAAGUGAAGAA & AACUUUACUUUAUUUAA	249.1	0	0.10

## Data Availability

The raw data resulting from sRNAseq (PRJNA764694), mRNAseq (PRJNA764690), and degradome sequencing (PRJNA1009842) were submitted to the NCBI SRA database.

## Supplementary Materials

The following supporting information can be downloaded at: https://www.mdpi.com/article/10.3390/ijms241915013/s1.
