# Supplementary material for: Combined Omics Approaches Reveal Distinct Mechanisms of Resistance and/or Susceptibility in Sugar Beet Double Haploid Genotypes at Early Stages of Beet Curly Top Virus Infection
Source: Int J Mol Sci. 2023 Oct 9;24(19):15013. doi: 10.3390/ijms241915013 (PMC10573692; doi:10.3390/ijms241915013)
Supplement: Supplementary file 1 [file ijms-24-15013-s001.zip › Figures. S1-S9.pdf]

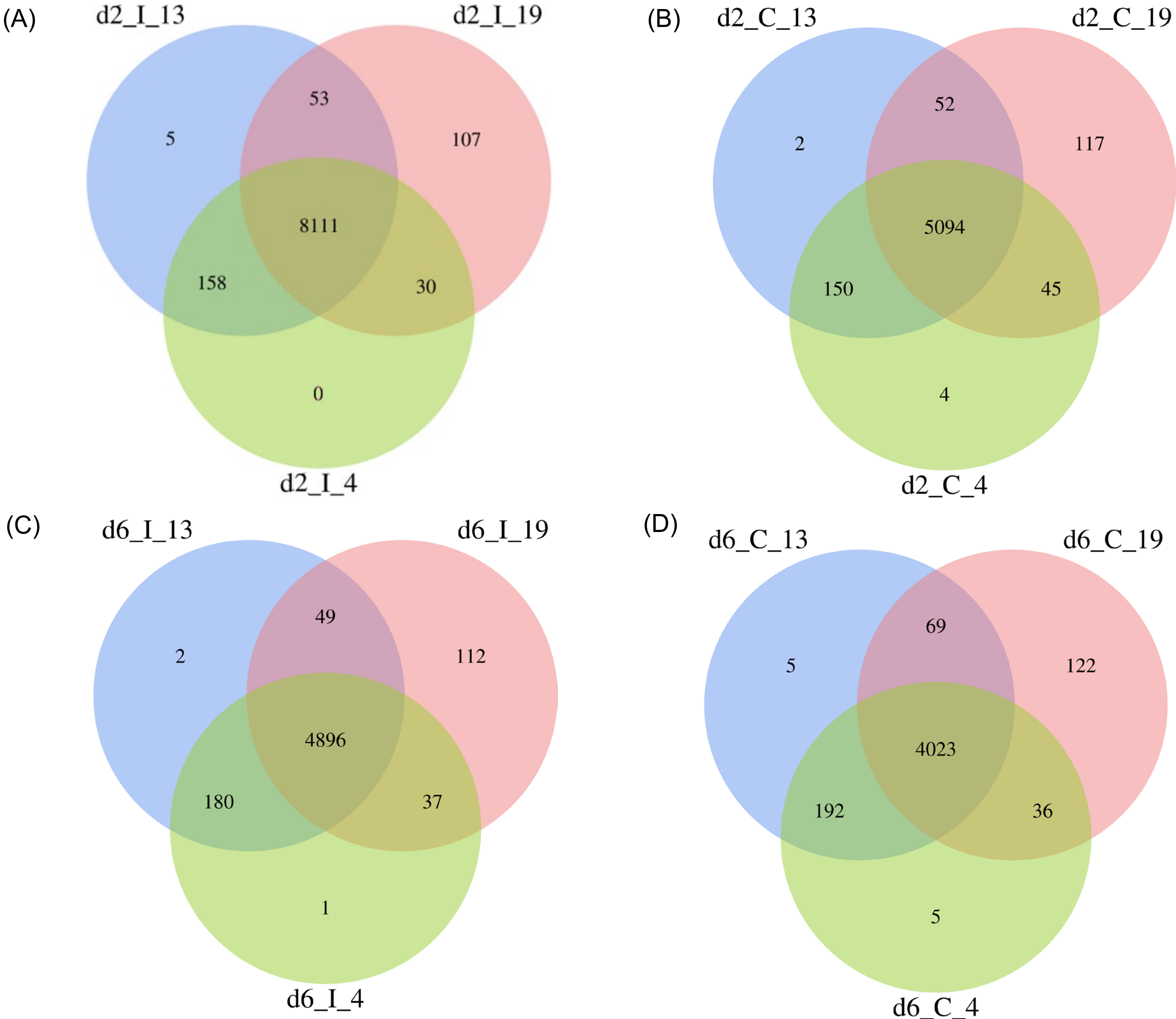

Figure S1. Venn diagram of differentially expressed (DE) sugar beet genes at 2 d and 6 d in the leaves of Beet curly top virus susceptible (Line 19; S) and resistant (Line 13 and Line 4; R) sugar beet lines infected and without virus infection. (A) 2 d infected (I), (B) 2 d uninfected control (C), (C) 6 d infected, and (D) 6 d uninfected control.

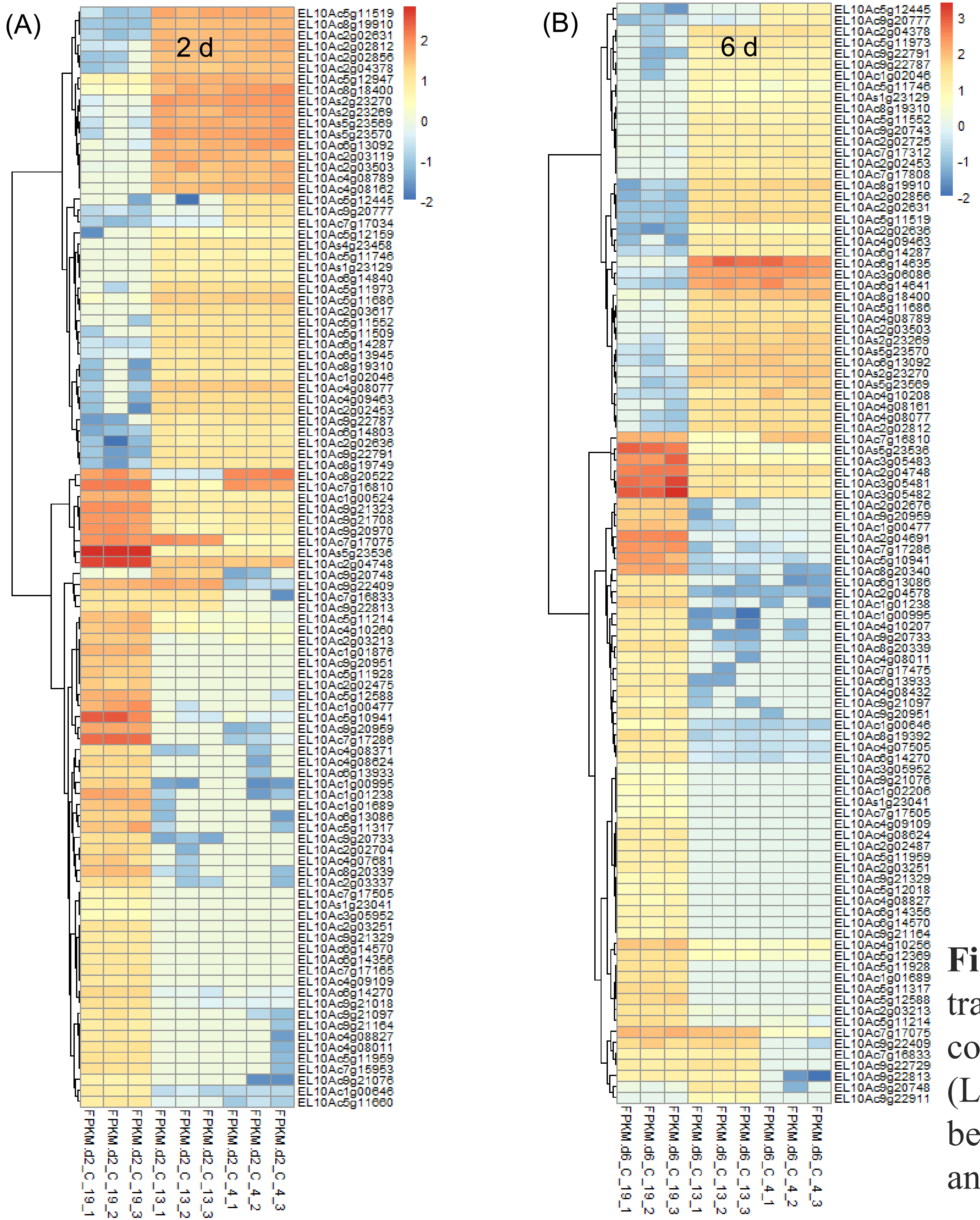

Figure S2. Heatmaps of differentially expressed (DE) transcripts at **(A)** 2 d control (C; uninfected), and **(B)** 6 d control (C; uninfected) in the leaves of BCTV susceptible (Line 19; S) and resistant (Line 13 and Line 4; R) sugar beet lines. Data are Mean ± SE of 4–5 biological replicates and $p \leq 0.01$ between ‘S’ and ‘R’ lines.

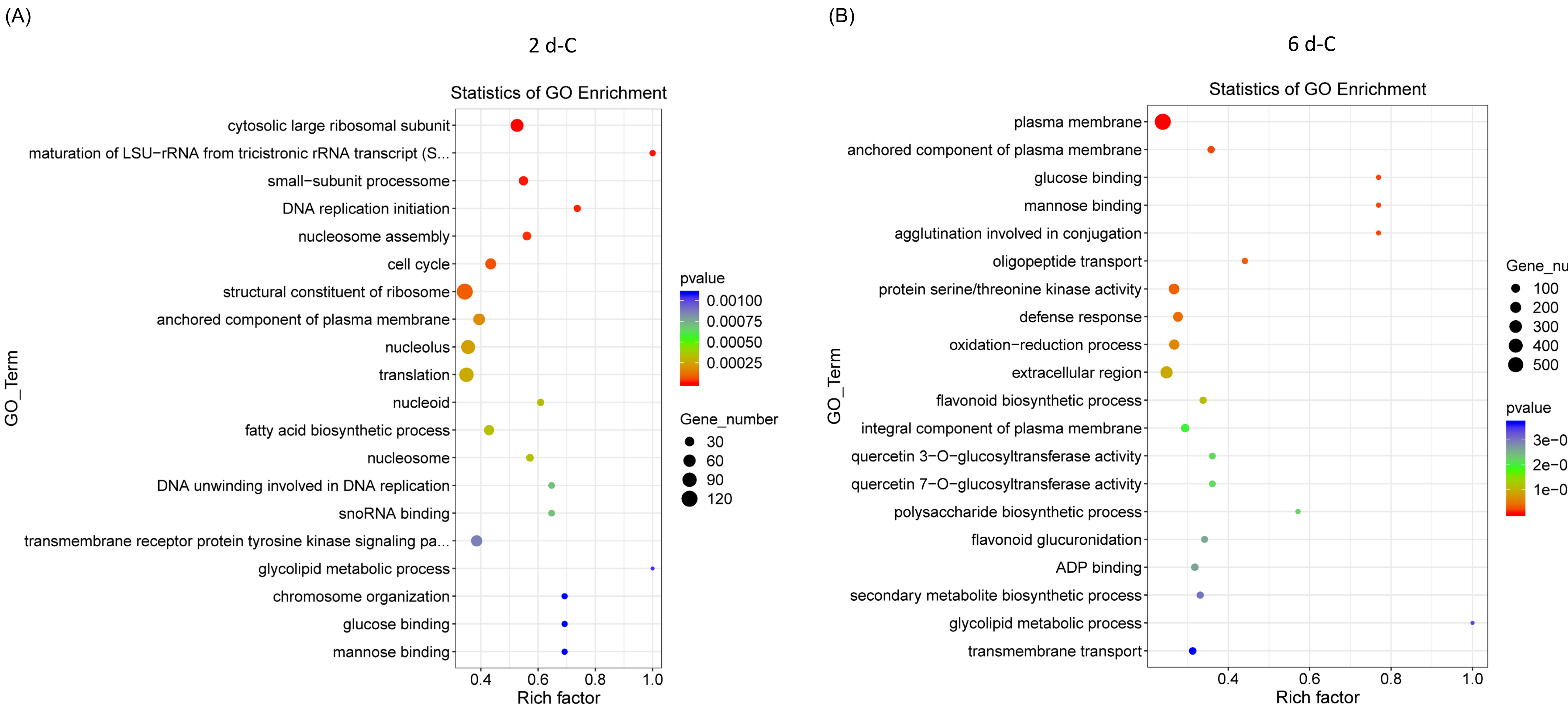

Figure S3. Gene ontology (GO) of differentially expressed sugar beet genes in the non-inoculated control (C) plants. (A) 2 d; and (B) 6 d. Data are mean of 3 biological replicates.

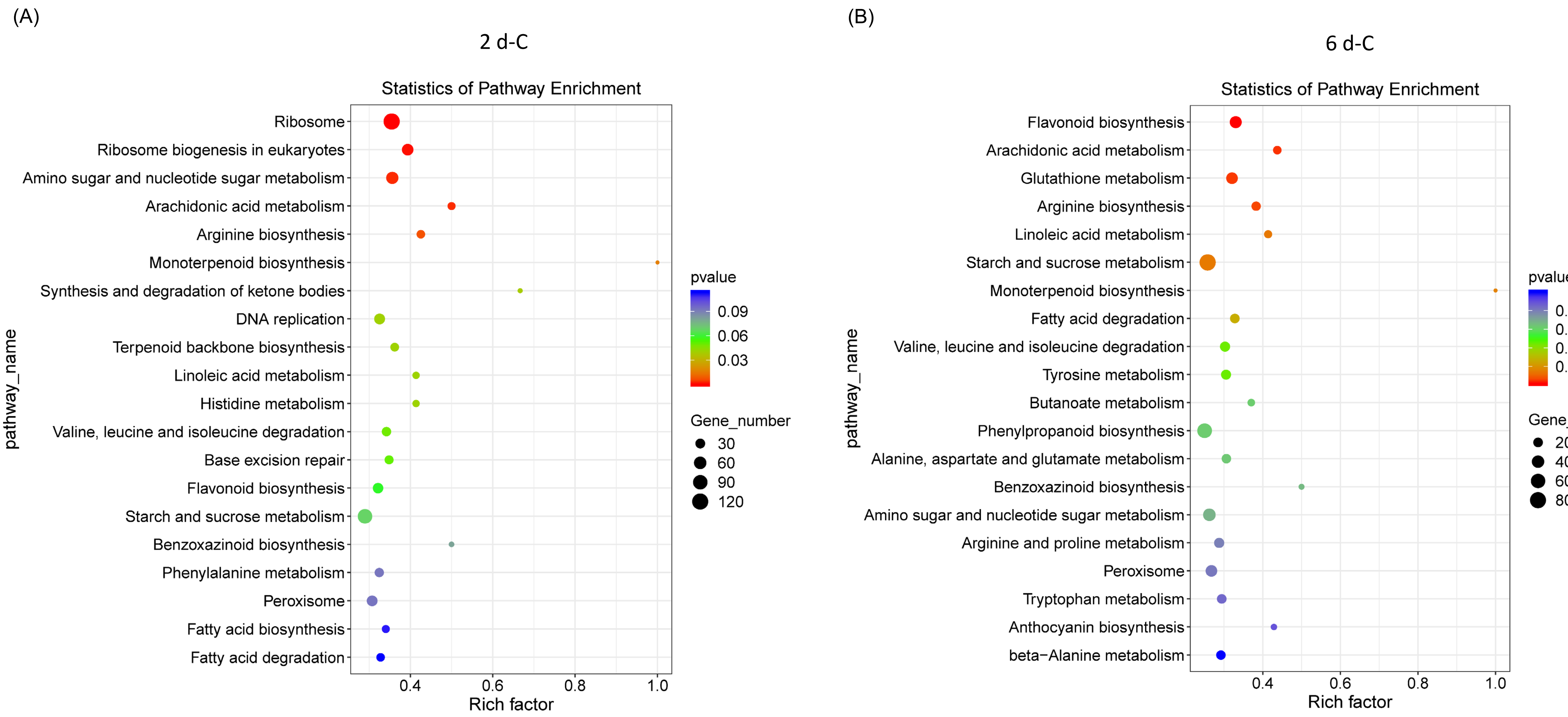

Figure S4. Pathway enrichment of differentially expressed sugar beet genes in the non-inoculated control (C) sugar beet plants. Kyoto Encyclopedia of Genes and Genomes (KEGG) enrichment of sugar beet genes in (A) 2 d, and (B) 6 d samples. Data are mean of 4-5 biological replicates.

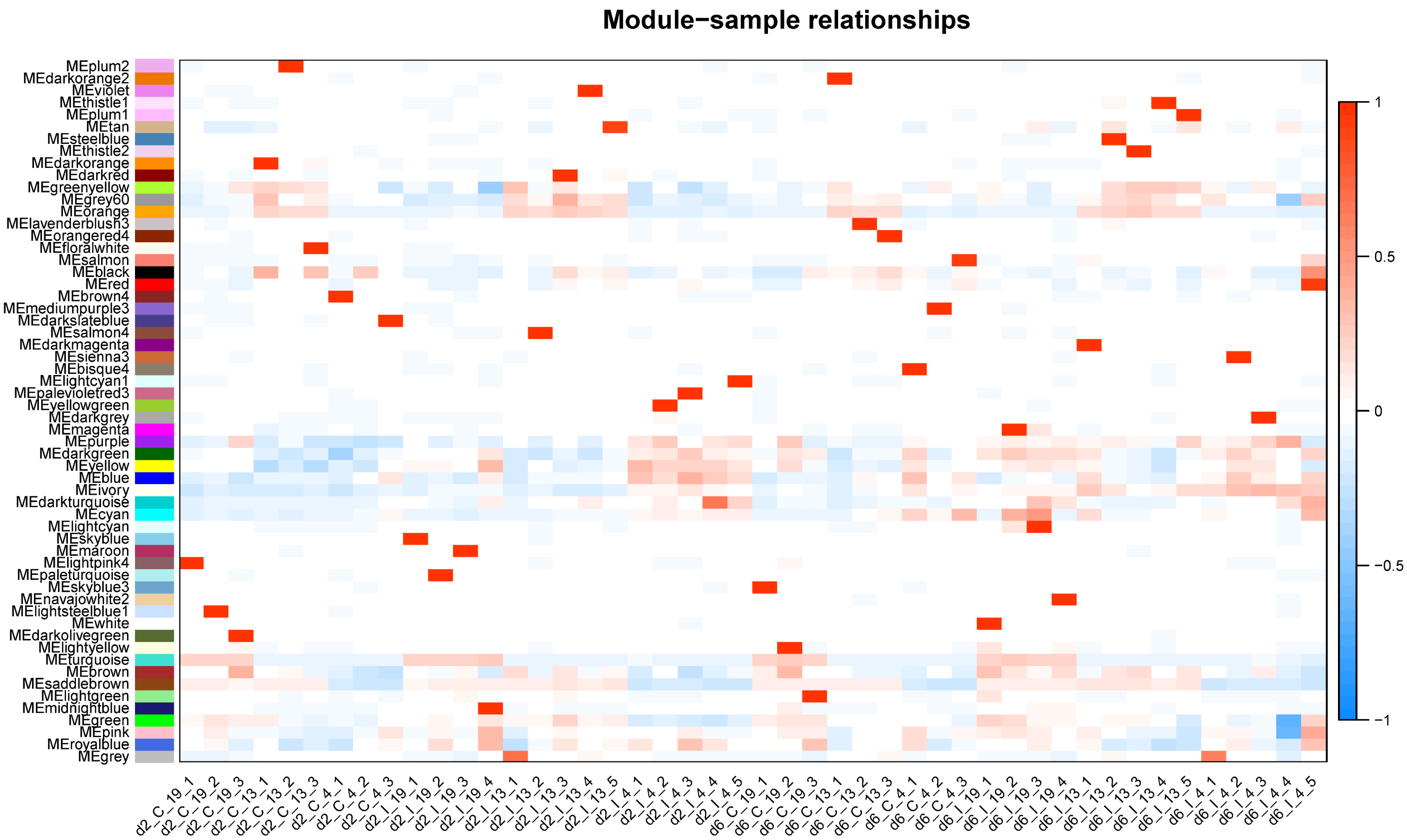

Figure S5. Weighted gene co-expression network analysis (WGCNA) of sugar beet genes in the leaves infected with (I) or without (C) Beet curly top virus (BCTV), show distinct clustering pattern (module-sample relationship) in the BCTV susceptible (Line 19; S) and resistant (Line 13 and Line 4) sugar beet lines.

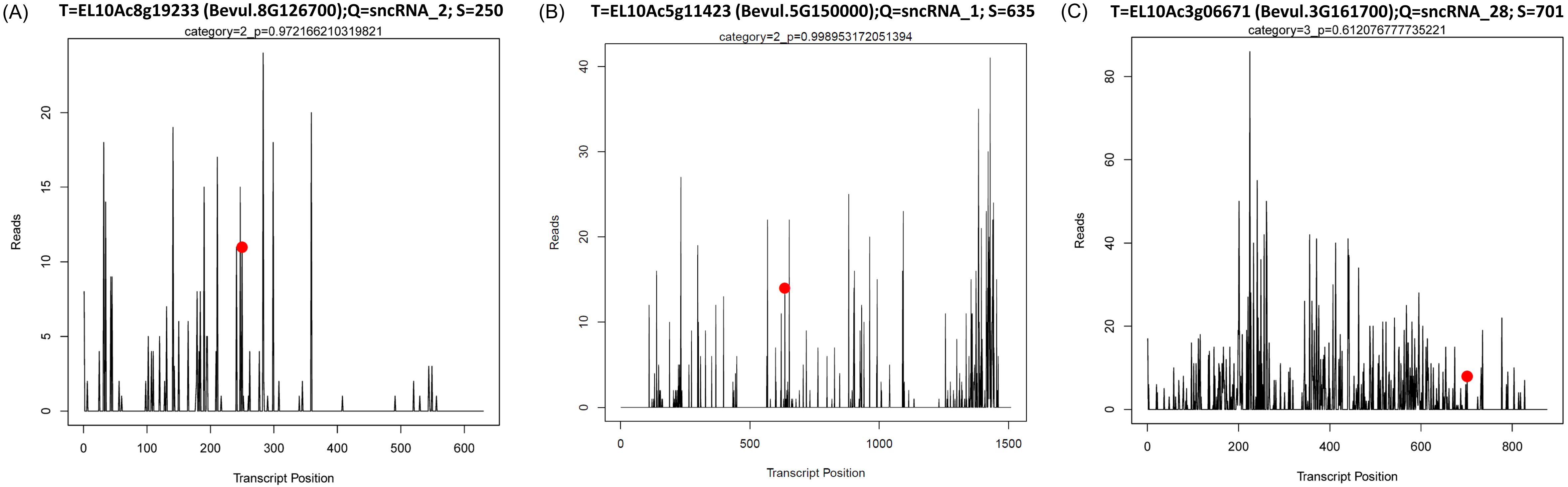

Figure S6. Target plot (t-plots) of Beet curly top virus (BCTV) derived representative small non-coding RNA (sncRNA) targets in different categories confirmed by degradome sequencing of the susceptible sugar beet Line 19 at 6 days post inoculation (dpi) leaf samples. (A) sncRNA_2, (B) sncRNA_1, and (C) sncRNA_28. The red dots denote putative cleavage site as predicted by the 'CleaveLand' pipeline [55]; T: sugar beet target transcript, Q: query BCTV sncRNA., S: nucleotide site of the cleavage.

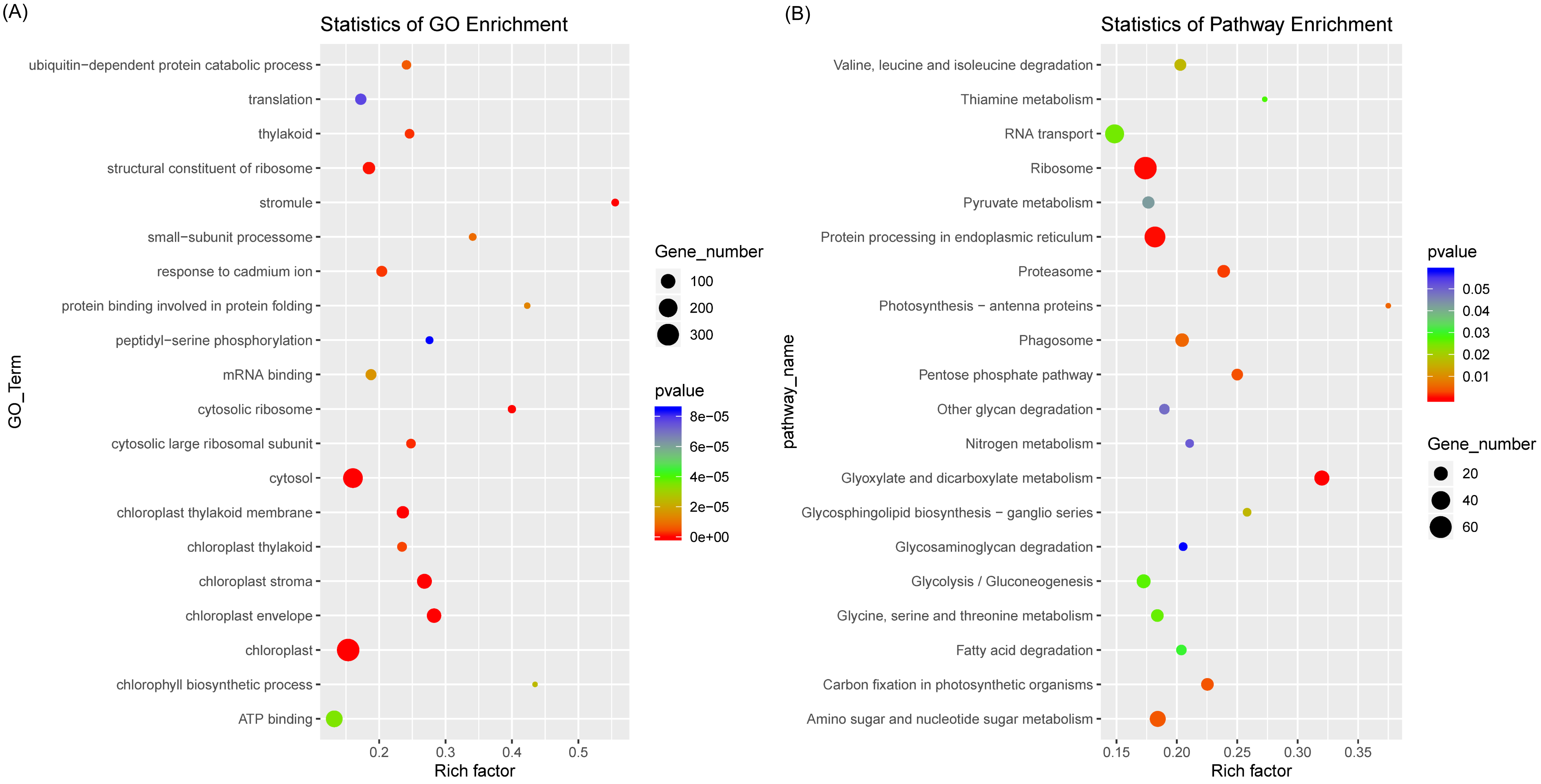

Figure S7. BCTV derived sncRNA target enrichment (based on degradome analysis) in the susceptible sugar beet Line 19 at 6 days post inoculation (dpi) samples. (A) Gene ontology (GO) enrichment, and (B) pathway enrichment.

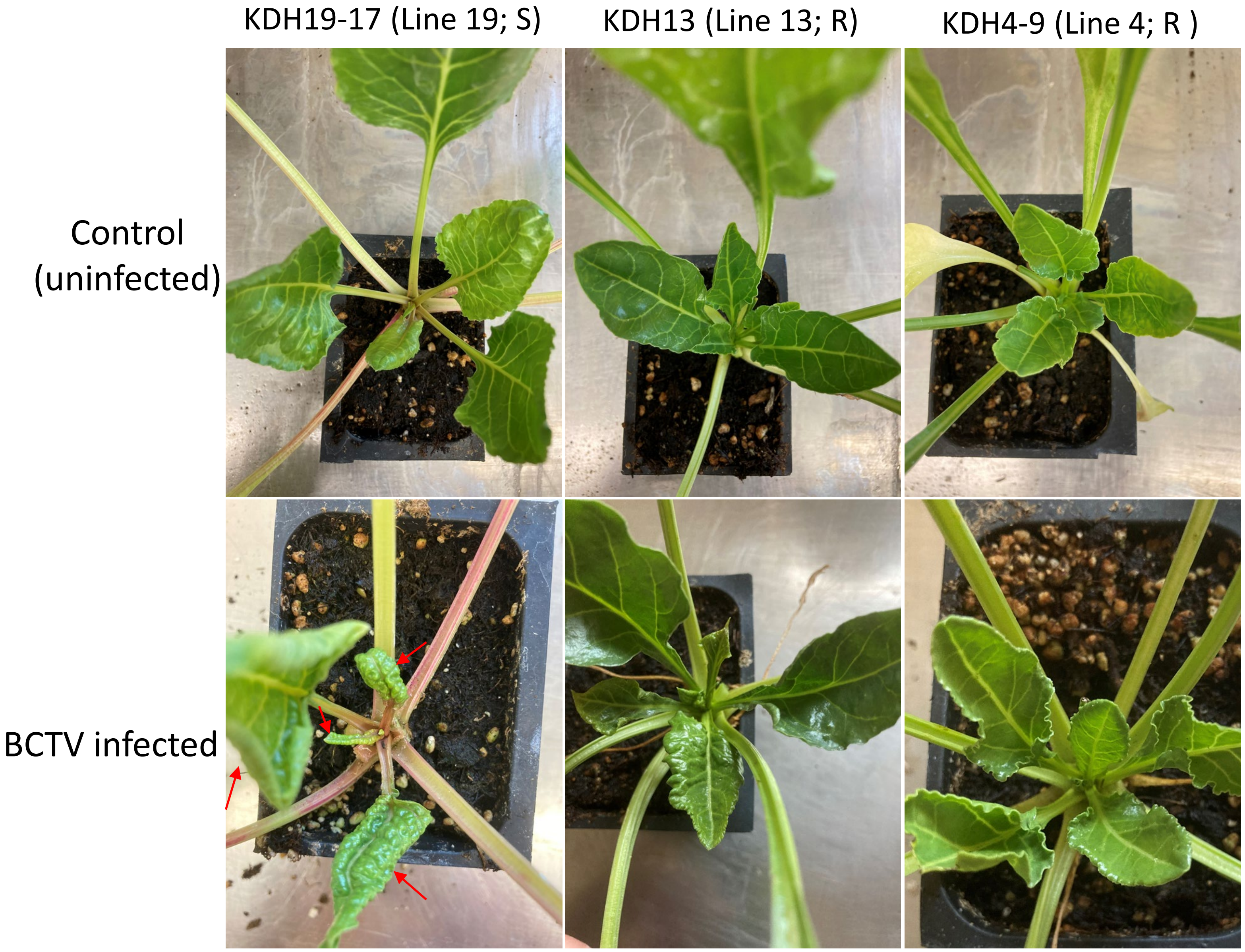

Figure S8. Disease symptoms (leaf curling and swelling) in the apical leaves (pointed by red arrows) of sugar beet genotypes at 3 weeks post inoculation (wpi). The upper panel shows phenotype of uninfected plants [susceptible (S); resistant (R)].

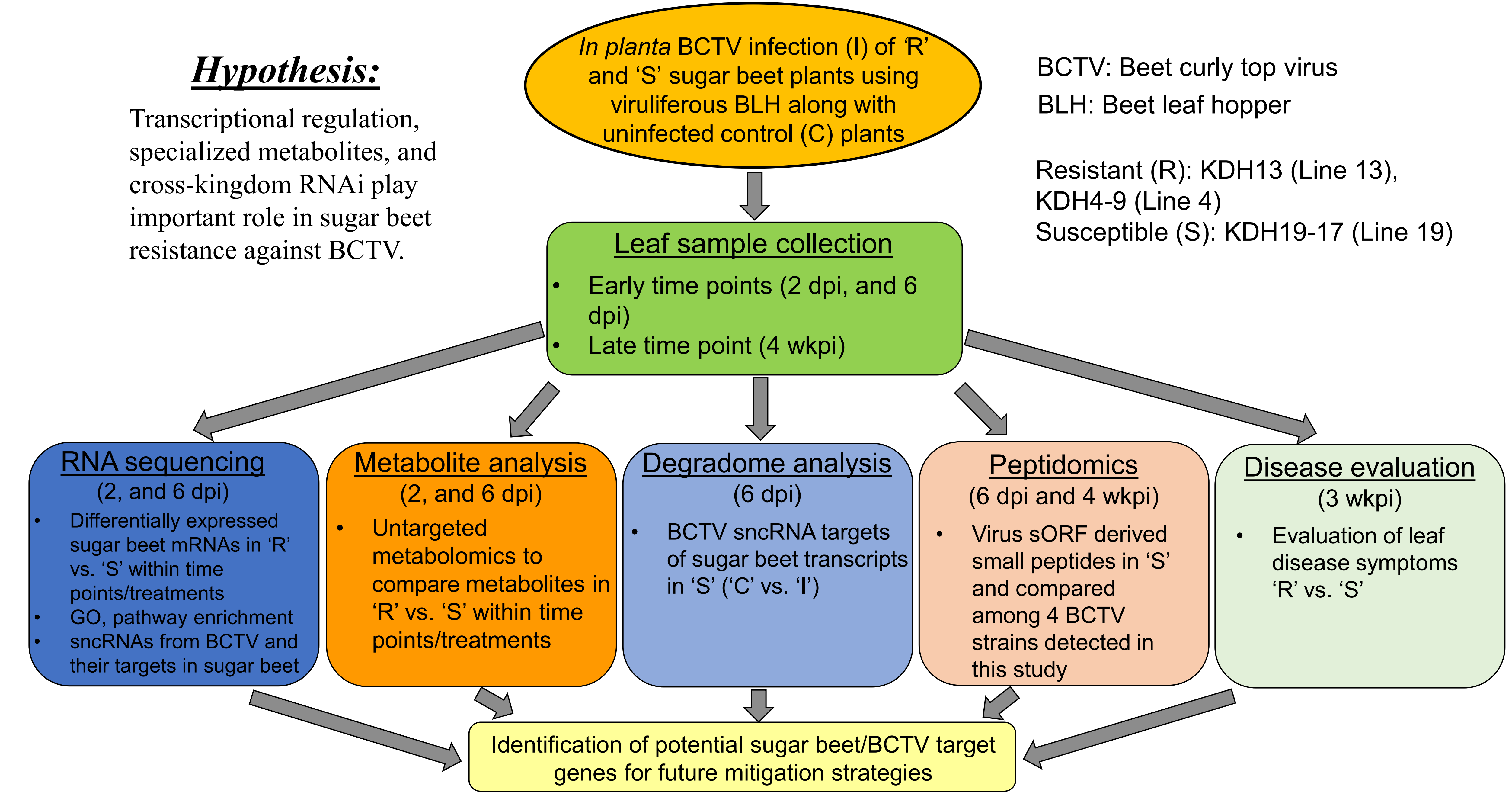

Figure S9. Experimental approach to delineate sugar beet resistance mechanisms against Beet curly top virus at early infection stages(dpi: days post infection; wkpi: weeks post infection; mRNA: messenger RNA; GO: gene ontology; sncRNA: small non-coding RNA; sORF: small open reading frame).
